# Biophysical Properties of Intrinsically Disordered p130Cas Substrate Domain — Implication in Mechanosensing

**DOI:** 10.1371/journal.pcbi.1003532

**Published:** 2014-04-10

**Authors:** Kinya Hotta, Soumya Ranganathan, Ruchuan Liu, Fei Wu, Hiroaki Machiyama, Rong Gao, Hiroaki Hirata, Neelesh Soni, Takashi Ohe, Christopher W. V. Hogue, M. S. Madhusudhan, Yasuhiro Sawada

**Affiliations:** 1 Department of Biological Sciences, National University of Singapore, Singapore; 2 School of Biosciences, The University of Nottingham Malaysia Campus, Selangor, Malaysia; 3 Mechanobiology Institute, National University of Singapore, Singapore; 4 Department of Physics, National University of Singapore, Singapore; 5 Indian Institute of Science Education and Research, Pune, India; 6 Locomotive Syndrome Research Institute, Nadogaya Hospital, Kashiwa, Japan; 7 Bioinformatics Institute, Singapore; Institut Pasteur, France

## Abstract

Mechanical stretch-induced tyrosine phosphorylation in the proline-rich 306-residue substrate domain (CasSD) of p130Cas (or BCAR1) has eluded an experimentally validated structural understanding. Cellular p130Cas tyrosine phosphorylation is shown to function in areas without internal actomyosin contractility, sensing force at the leading edge of cell migration. Circular dichroism shows CasSD is intrinsically disordered with dominant polyproline type II conformations. Strongly conserved in placental mammals, the proline-rich sequence exhibits a pseudo-repeat unit with variation hotspots 2–9 residues before substrate tyrosine residues. Atomic-force microscopy pulling experiments show CasSD requires minimal extension force and exhibits infrequent, random regions of weak stability. Proteolysis, light scattering and ultracentrifugation results show that a monomeric intrinsically disordered form persists for CasSD in solution with an expanded hydrodynamic radius. All-atom 3D conformer sampling with the TraDES package yields ensembles in agreement with experiment when coil-biased sampling is used, matching the experimental radius of gyration. Increasing β-sampling propensities increases the number of prolate conformers. Combining the results, we conclude that CasSD has no stable compact structure and is unlikely to efficiently autoinhibit phosphorylation. Taking into consideration the structural propensity of CasSD and the fact that it is known to bind to LIM domains, we propose a model of how CasSD and LIM domain family of transcription factor proteins may function together to regulate phosphorylation of CasSD and effect machanosensing.

## Author Summary

Mechanical stretching of cells causes the substrate domain of p130Cas (CasSD) to be phosphorylated on 15 tyrosine residues embedded along its length. CasSD is rich in proline and surprisingly well conserved in placental mammals. Stretching of CasSD by atomic force microscopy has identified that it requires far less force than normal folded proteins. Classical biophysical analyses have determined that CasSD is a typical intrinsically disordered protein, a difficult-to-study group of molecules covering about 30% of human proteins. The average size of CasSD is larger and elongated than folded globular proteins but smaller than chemically denatured proteins. We have simulated a large number of all-atom protein structures using a fast all-atom sampling method. The result is in good agreement with the experimental observation. As it is already known that stretching somehow exposes the tyrosine residues to phosphorylation, a mechanism is proposed where straightening of the p130Cas substrate domain backbone conformation through mechanical stretching can lead to dissociation of p130Cas-binding LIM domain proteins and exposure of CasSD tyrosine residues for phosphorylation. This study has led to a new model of a protein-based mechanism of force sensing at the leading edge of cells that allows the cells to feel their way as they move.

## Introduction

**Figure 1:**
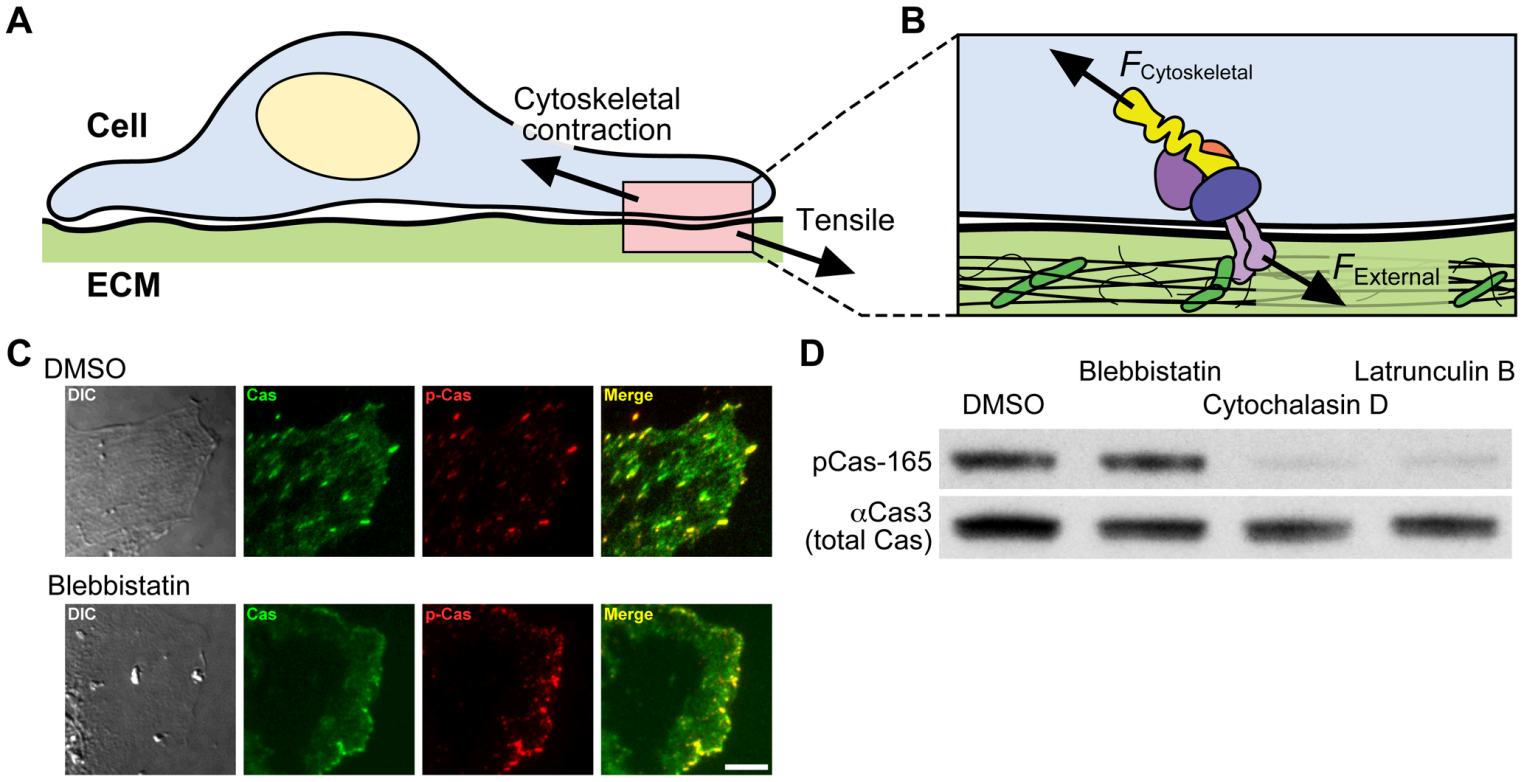
Phosphorylation of p130Cas does not depend on actomyosin contractility. (A) Scheme showing a cell experiencing a tensile force from the extracellular matrix (ECM). (B) Close-up view of the molecular interactions occurring at the cell–ECM junction. Proteins associated with the adhesions (multi-colored circles) and a mechanosensing protein (yellow) are shown as stretched by the inward-facing cytoskeletal contraction forces (*F*_Cytoskeletal_) and the external tensile forces (*F*_External_). (C) p130Cas-deficient fibroblasts expressing GFP–p130Cas in the presence (the “Blebbistatin” row) or absence (the “DMSO” row) of 10 µM blebbistatin were viewed with a TIRF microscope. Differential interference contrast (DIC, first column), GFP TIRF (Cas, second column), and Alexa546 TIRF (p-Cas, third column) images of a leading cell in scratched monolayer were acquired. Merged images of GFP and Alexa546 (Merge, fourth column) are shown. Scale bar: 10 µm. (D) HEK293 cells cultured on collagen-coated substrate were treated with DMSO (0.1%), blebbistatin (50 µM), cytochalasin D (0.5 µM) or latrunculin B (0.5 µM) for 30 minutes. After treatment, the cells were lysed and equivalent amounts of cell lysates were subjected to SDS-PAGE followed by western blot analysis using anti-phospho-p130Cas-Y165 (pCas-165) and anti-p130Cas (αCas3) antibody.

p130Cas (mouse: NP_001185768; rat: NP_037063) is a proline-rich scaffold protein that plays an essential role in various cell functions, including motility [1], survival [2], apoptosis [3] and transformation [4]. The substrate domain, CasSD, is centrally located and contains 15 repeats of YxxP motifs that can be a substrate of Src family kinases [5]. Tyrosine phosphorylation of the CasSD YxxP motifs creates binding sites for the SH2 and PTB domains of effector signaling proteins, such as Crk and Nck. The presence of other domains in p130Cas, namely the N-terminal SH3 domain, the serine-rich domain and the C-terminal Src-binding domain, also allow p130Cas to interact with various other signaling molecules, including focal adhesion kinase (FAK), 14-3-3 proteins and Src family kinases. The ability of p130Cas to associate with a large array of signaling proteins appears to facilitate the formation of multi-protein complexes that allow protein–protein interactions among the bound molecules to promote effective transduction of cellular signals [6]. Various growth factors, hormones, and integrin-mediated adhesion have been reported to regulate tyrosine phosphorylation of CasSD. For example, activation of receptor protein tyrosine kinases by growth factors [7], activation of estrogen receptor via estrogen binding [8], or direct interaction between integrin and FAK [9] result in activation of Src and FAK, leading to phosphorylation of tyrosine residues within CasSD. Of those, the most intriguing function that is assigned to p130Cas is its ability to act as a force sensor. We previously demonstrated that physical stretching of CasSD renders it susceptible to phosphorylation of its tyrosine residues by Src family kinases [10]. The multiply phosphorylated CasSD can then act as a docking site for a variety of signaling molecules as described earlier. Evidence from a variety of methods also exists that shows that the LIM domain proteins zyxin [11] and TRIP6 [12], [13] bind to unphosphorylated p130Cas, localized to sequence within CasSD, and requiring at least 2 LIM domain repeats for binding. In cells, p130Cas can localize to focal adhesions by interacting with FAK through its N-terminal SH3 domain [14]. Since focal adhesions are where FAK associates with actin cytoskeletons via talin [15], we postulated that extension of p130Cas depends on the tensile forces generated between actin cytoskeletons and cell–extracellular matrix (ECM) contacts (Figure 1A and B) [10]. By transforming a mechanical event that occurs at a cell-stretching site into a tyrosine phosphorylation signal, p130Cas can act effectively as a cellular mechanosensor. However, the details of the strength of the type of cell-generated forces that stretch CasSD and facilitate its phosphorylation have remained poorly defined. In addition, structural mechanism underlying the responsiveness of CasSD to mechanical stretching is yet to be determined. Since structural information would be critical in understanding how the conformational change of CasSD can occur in response to a tensile force, we set out to determine the biophysical and structural properties of CasSD using a combination of various *in vivo, in vitro* and *in silico* characterization techniques.

Proline accounts for 19.9% (61 out of 306 residues) and 20.9% (64 out of 306 residues) of the composition of mouse and rat CasSD, respectively. We therefore anticipated, and demonstrate herein, that CasSD is an intrinsically disordered domain (IDD). There are many intrinsically disordered proteins (IDP) found in nature [16], but only a few IDDs have undergone intense structural scrutiny. Several of these IDD-containing proteins are known scaffold proteins [17]–[20]. Just as CasSD has been known to interact with several different protein partners, it has been noted that IDDs also associate with promiscuous interacting partners and often form hubs of interactions networks [21]–[25]. While there is clearly no apparent single low-energy folded structure in uncomplexed IDDs [26], advancements in experimental and computational approaches have allowed better characterization of ensemble states and insight into local polypeptide backbone conformational preferences. A growing consensus suggests that the normal peptide backbone angle distribution of IDDs contains a large number of PPII conformations [27]–[29], except in those instances where there is some evolutionary conservation of protein-fold sequence as in the SH3-like DRK IDD [30], or where there are local regions of strong α-helical propensity, such as those found in the N_TAIL_ protein of measles and related viruses [31]. In NMR studies of IDDs, the consensus approach to working with ensemble information has been to generate large numbers of candidate structures using various software systems that sample conformational space [32]–[34], and then remove those structures that are excluded by a variety of measurable constraints [30], [35]. The NMR fitting of several IDPs has provided a general knowledge that IDD sequences have a natural propensity to sample from PPII conformations [31], while chemical or thermal denaturation alters this propensity more toward unpaired β-strand type dihedral angle conformations [29]. The Trajectory Directed Ensemble Sampling package (TraDES-2, http://trades.blueprint.org) [32] has been used for generating conformational space samples in some of these studies. It employs brute-force sampling of protein conformations to search for fully folded structures and for creating ensembles of conformations for disordered proteins. In principle, the method could constrain the conformations according to given experimental data. However, no such constraints were used in this study. Taking this new information about PPII conformational sampling propensity into account, we set out to create large ensembles of plausible all-atom 3D structures of CasSD with varying amounts of PPII and β bias, and compare the polymer properties of this *in silico* ensemble with similar ensembles made with increasing amounts of unpaired β-strand dihedral conformations. We then compared the computed polymer properties of three separate ensembles to those measured by a variety of biophysical techniques to determine whether a coil (PPII biased) ensemble can recapitulate the experimental parameters we have determined.

## Results

### Phosphorylation of p130Cas in adherent cells depends upon actin polymerization, but not actomyosin contraction

p130Cas is phosphorylated at cell–matrix contact sites (focal adhesions) where cytoskeletal tensile force is transmitted to ECM (Figure 1A and B) [36]. Since cell stretching is thought to increase the tensile force exerted on the molecules localized at the sites of ECM–cytoskeleton linkage [10], we initially speculated that phosphorylation of p130Cas molecules at adhesion sites would depend on the contractility of actin cytoskeletons derived from myosin motor activity [37]. Cells exert centripetal traction forces on substrate to which they adhere, even while stretching forces are not externally applied [36]. We therefore expected that inhibition of myosin II would decrease stretching forces on p130Cas at focal adhesions and thereby affect its phosphorylation. Contrary to this notion, p130Cas exhibited distinct phosphorylation at the leading edge of migrating cells even when cells were treated with a myosin II inhibitor, blebbistatin (Figure 1C). Furthermore, when we treated spread NIH3T3 cells with blebbistatin, we found that p130Cas phosphorylation was not significantly decreased (Figure 1D). These results indicated that p130Cas phosphorylation does not depend upon actomyosin contractility. In contrast, inhibition of actin polymerization by cytochalasin D or latrunculin B greatly attenuated the phosphorylation of p130Cas (Figure 1D). Since p130Cas is phosphorylated at the leading edge of migrating cells where actin is actively polymerized independently of myosin II activity (Figure 1A and B) [38], these results suggested that CasSD may be stretched for phosphorylation by the force generated by actin polymerization (∼5 pN), which would be significantly weaker than the actomyosin-generated force (∼30 pN per integrin bond) [39].

### Determination of force required to unfold CasSD by atomic-force microscopy

**Figure 2:**
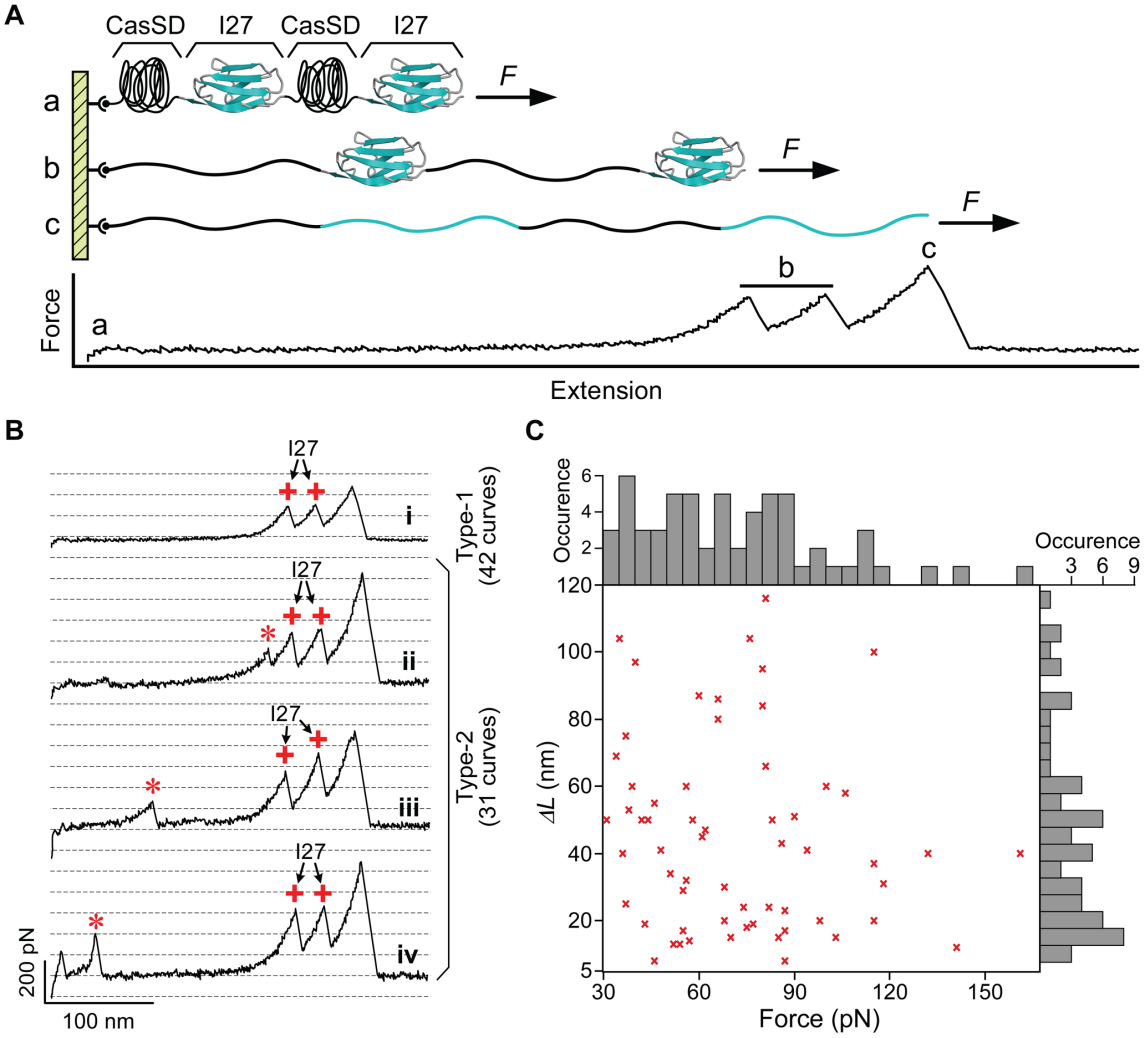
AFM stretching of individual CasSD–I27–CasSD–I27 molecules in a constant-velocity mode. (A) Scheme of the CasSD–I27–CasSD–I27 protein construct used in the current study where it is stretched from the two termini. (B) Typical force vs. extension trajectories obtained. Only the curves showing signatures of two I27 unfolding peaks (*ΔL* = 28±2 nm, F>100 pN, labeled +) are recorded (73 traces), and any other feature (labeled *) not associated with I27 is assigned to CasSD domains. Curve *i* (type-1), a typical trajectory with only two force peaks for I27, indicating that this CasSD has a floppy structure with almost no resistance to AFM pulling (below the detection limit 15 pN); Curve *ii* to *iv* (type-2), typical trajectories with some features for CasSD. Type-1 curves (42 traces) dominate. (C) A plot of unfolding peak forces vs. contour length changes *ΔL* from type-2 curves as shown in (A) (I27 excluded), showing points distributed without correlation between peak forces and *ΔL* for all unfolding events. Top and right side panels of (C) show broadly and randomly distributed histograms of peak force and *ΔL* respectively.

To analyze the mechanical stability of CasSD, single-molecule force measurements by atomic-force microscopy (AFM) in a constant-velocity mode have been carried out on a protein construct CasSD–I27–CasSD–I27, where two I27 domains are introduced as referenced unfolding signature. I27 domain was used, because the elastic property of this domain has been well characterized [40], and its good mechanical strength makes it easy to be identified from other proteins [41]. Though a hexahistidine (His_6_)-tag is introduced at the N-terminal of the construct and nickel-nitrilotriacetic acid (Ni-NTA) on substrate surfaces to promote the binding of protein molecules to the substrate surface at its N-terminal (Figure 2A), it is still possible to pull a molecule from any two points along its length in the actual experiment. However, the two I27 domains will always have one CasSD domain in between them as shown by the scheme in Figure 2B. Once an I27 domain unfolds (Figure 2A parts b and c), a signature force peak will be recorded on the force–extension trajectory (peaks labeled b in the bottom panel of Figure 2A). Thus, we can be certain that at least one CasSD domain is stretched in trails that show two force peaks for I27 domains in the force-extension trajectories. Any peaks other than the two I27 peaks in those trajectories would be considered as the signal from stretching any mechanically stable structure associated with CasSD.

Out of 73 curves obtained with two identified force peaks for I27, there were 42 curves (type-1) that showed no other distinct feature as shown in Figure 2B, curve *i*. This indicates that the CasSD domains stretched in these trials consist of only floppy structures with limited mechanical strength that cannot be detected by AFM (<15 pN). Because a fast pulling speed of 600 nm/s was used in these AFM measurements, the unfolding force of CasSD *in vivo* is expected to be even smaller. The rest of the trajectories (type-2) did show some features (Figure 2B, curves *ii*–*iv*) other than I27's. Both *F*_unfold_ (Figure 2C, top side panel) and *ΔL* (Figure 2C, right side panel) were broadly distributed, ranging from 30 to 120 pN and from 5 to 120 nm, respectively. The unfolding peak force *F_unfold_* and contour length change *ΔL* showed no correlations since no dominant region can be found in Figure 2C. The relationship between *F_unfold_* and *ΔL* as well as their distributions indicate that within those CasSDs showing type-2 curves, only random structures with random mechanical strength are found. Therefore, results from single-molecule force measurements suggest that the structure of CasSD is predominantly random and flexible. The variability of the pull distance of CasSD indicates significant variation among structures of CasSD, which may be related to its fundamental function as a reporter of subtle mechanical transformations in its environment. Importantly, most structures of CasSD possess little mechanical stability, implying that CasSD can be stretched readily with the weak force generated by actin polymerization. This unexpected mechanical flexibility of CasSD requires modification to the stretch-sensor model illustrated in Figure 1B that involves stretching of CasSD with much stronger tensile forces derived from actomyosin contractility. To gain better understanding of the structural basis of this intrinsic mechanical flexibility of CasSD, further biophysical analyses and simulations were undertaken.

### Biophysical characterization identifies CasSD as intrinsically disordered and monomeric

To obtain a large-scale preparation of a purified protein for further structural characterizations, CasSD was expressed as a tobacco etch virus (TEV) protease-cleavable C-terminal His_6_-tagged protein in *E. coli* BL21(DE3). We also employed recombinant rat CasSD for some of our experiments, because a method for faster and higher yielding CasSD production became available. The purified CasSD was analyzed by sodium dodecyl sulfate–polyacrylamide gel electrophoresis (SDS–PAGE) to confirm that the sample was at least 95% pure (Figures S1A and S3A for mouse and rat CasSD, respectively). While we expected that mouse CasSD and rat CasSD would behave in a virtually identical manner because of their high overall amino acid sequence homology (96.4% identity and 97.4% similarity), we confirmed this by comparing their profiles during the SDS–PAGE (Figure S1A vs. S3A), SEC (Figure S3B) and circular dichroism (Figure S3C) analyses. The first indication of CasSD being an IDD was observed during SDS–PAGE analysis, where the protein anomalously migrated at 15–20% larger than the molecular weight determined by mass spectrometry (Figure S1B). This is a typical behavior of polar-than-normal IDPs, which bind less SDS and hence migrate more slowly than typical protein molecules [21]. Another hallmark characteristics of IDP is their elevated susceptibility to degradation by proteases [42]. CasSD was readily degraded by limited proteolysis using trypsin at 1∶2000 mass ratio to CasSD at a low reaction temperature (i.e., on ice) (Figure S2). Proteolytic degradation crudely indicates that, like other IDPs, CasSD does not assume a tightly folded structure. Those initial observations indicated that CasSD is likely an IDD.

**Figure 3:**
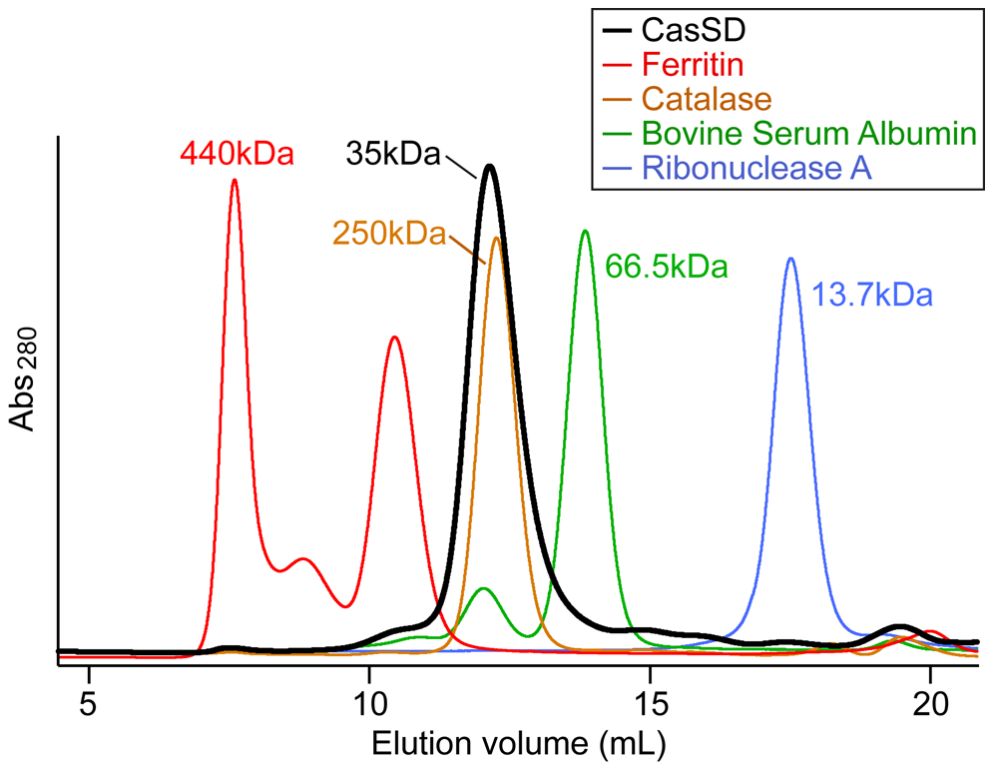
Characterization of CasSD by analytical size-exclusion chromatography. Purified CasSD was subjected to an analytical size-exclusion chromatography. Sample buffer and running buffer used were 10 mM potassium phosphate at pH 7.5, 100 mM potassium chloride, 1 mM EDTA and 5% (v/v) glycerol. Reference proteins were also subjected to the same chromatographic treatment for comparison. Red: ferritin; orange: catalase tetramer; green: bovine serum albumin; blue: ribonuclease A. Ferritin assumes a 440-kDa complex comprised of 24 subunits of light and heavy chains. Peaks eluting at a higher elution volume are thought to be ferritin molecules comprised of fewer subunits.

**Figure 4:**
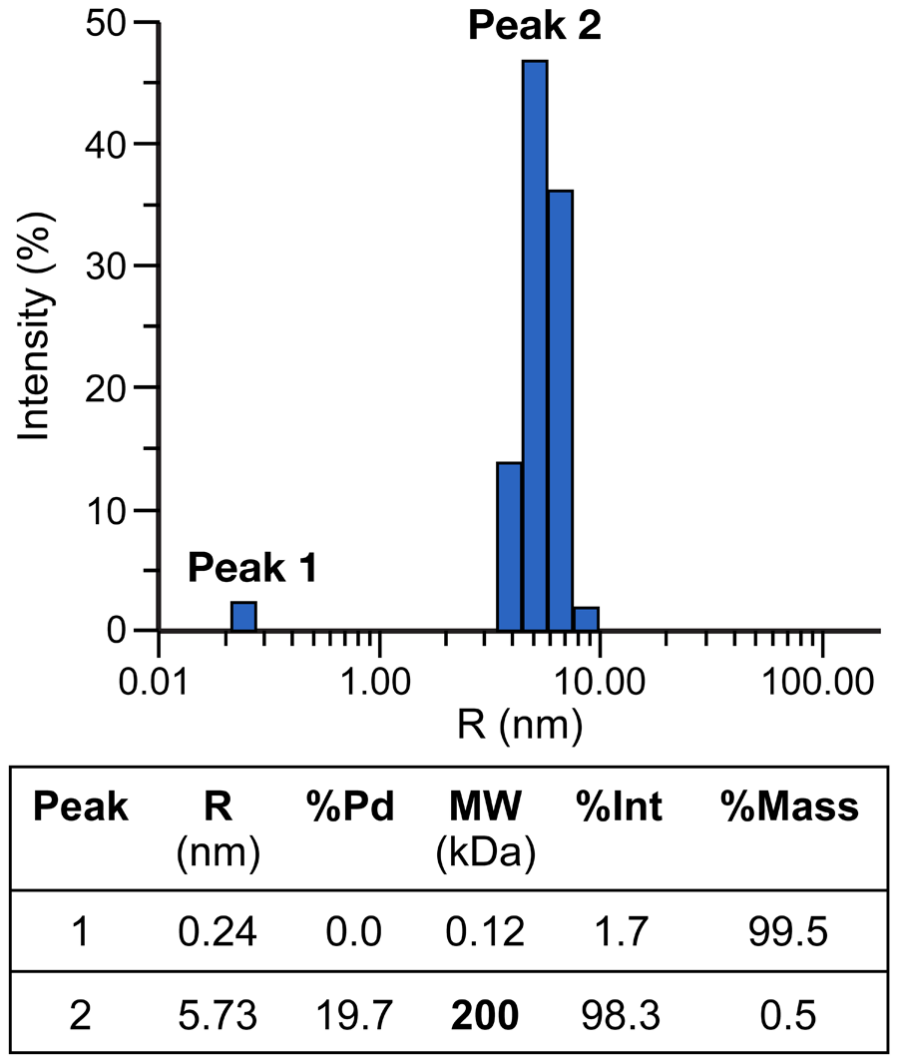
Dynamic light scattering measurements of purified CasSD. Diffusion coefficient, hydrodynamic radius, molecular weight and polydispersity of CasSD were calculated from the measurements. CasSD was in 10% (v/v) glycerol. Measurements collected at CasSD concentrations raging from 1 to 3 mg/mL were similar to each other.

**Figure 5:**
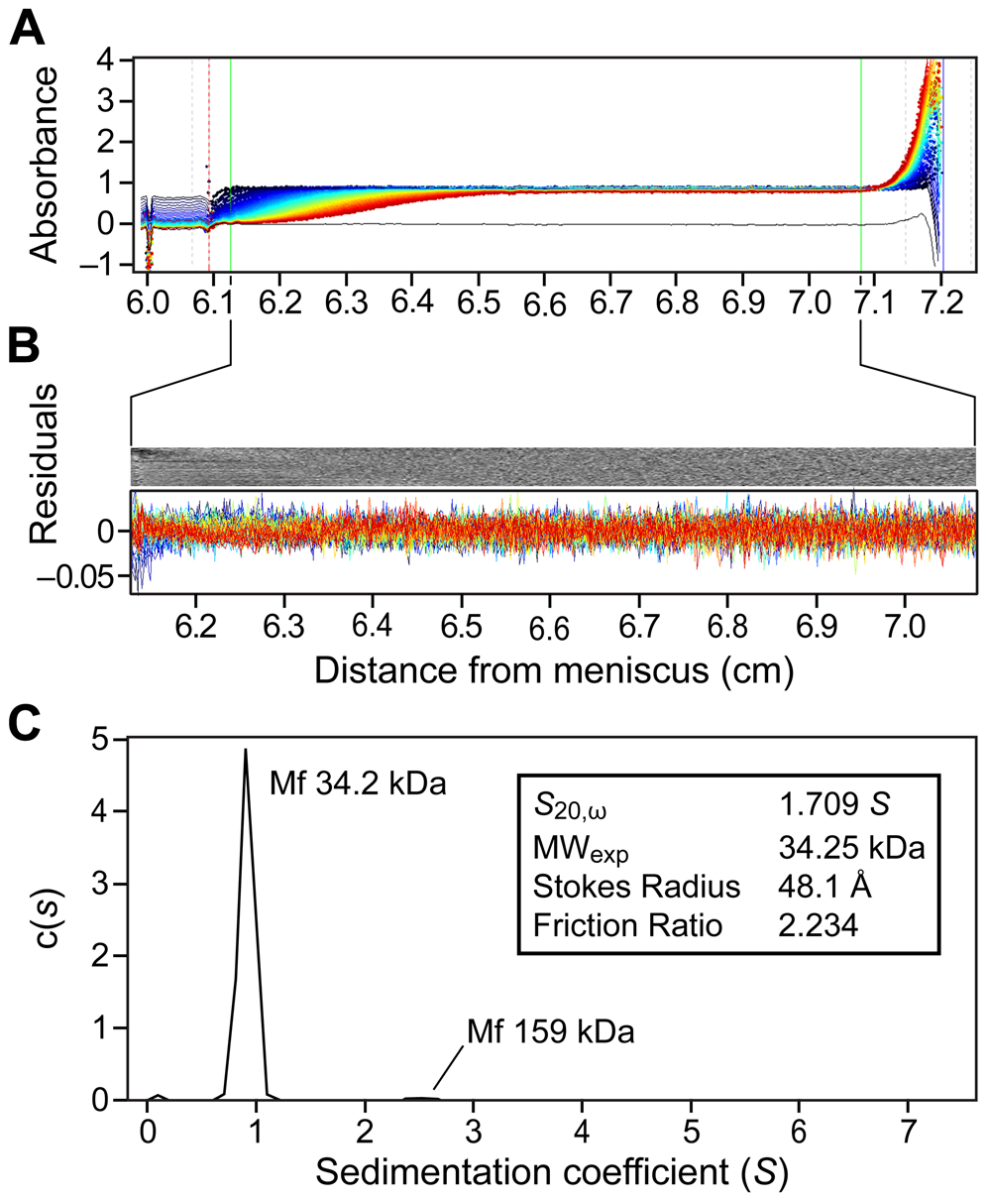
Sedimentation velocity analytical ultracentrifugation of purified CasSD. CasSD was subjected to analytical ultracentrifugation experiment in 10% (v/v) glycerol at a concentration of 1 mg/mL at 4°C over 7.3 hours. Data was fit to the continuous distribution model to obtain the experimental molecular weight, Stokes radius and frictional ratio of CasSD. (A) Raw data from the time-course measurement of absorbance of the sample at 280 nm along the sample cell length. (B) Residuals after fitting the data to the continuous size-distribution model. (C) Sedimentation coefficient distributions c(s) calculated from the sedimentation profile showing the calculated parameters in the inset.

To gain better understanding of the unique structural property of CasSD, we applied various analytical techniques to the purified recombinant CasSD. When we performed an analytical SEC experiment to examine the hydrodynamic property of CasSD, we found that CasSD clearly behaved as a single, homogeneous species (Figure 3) but with a broader peak width than standards. When compared to the standard reference proteins, CasSD was eluted from the column much earlier than a typical monomeric globular 35-kDa protein. Based on the chromatograms obtained for the reference proteins, the apparent molecular weight of CasSD based on the elution volume can be estimated to be close to a bovine catalase tetramer, which has a molecular weight of 250 kDa and Stokes radius of 51.2 Å [43]. The peak breadth may have arisen from conformational heterogeneity in the sample. Dynamic light scattering (DLS) was also measured to obtain additional information on the hydrodynamic property of CasSD obtained based on a different physical principle employed in SEC, where the outcome can be biased by ionic interactions between the sample and the matrix. DLS indicated that CasSD exhibits a monomodal, reasonably monodispersed distribution in a neutral potassium phosphate buffer with an apparent molecular weight of around 200 kDa (Figure 4), a result that is in agreement with the results from the SEC experiment. Those results suggest that CasSD assumes a shape that deviates from a typical globular protein to give an apparent molecular weight that is significantly larger than its calculated monomeric molecular weight. However, neither technique could directly distinguish whether the observed large molecular weight was due to an oligomer or a non-globular structure. In order to address this, we employed the sedimentation velocity analytical ultracentrifugation (SV-AUC) technique to characterize CasSD (Figure 5). SV-AUC on the purified CasSD allowed determination of its experimental molecular weight to be 34.2 kDa. With the calculated molecular weight of 34.9 kDa, this result confirms that CasSD exists as a monomer in solution. SV-AUC also allows calculation of the Stokes radius of the sample, which represents the hydrodynamic radius (*R*_H_) of a protein molecule. From the SV-AUC data, the *R*_H_ of CasSD was calculated to be 48.1 Å, which is in agreement with the SEC result. Since the minimal *R*_H_ of an ideal protein sphere with a molecular weight of 34.9 kDa is calculated to be 21.6 Å [44], the friction ratio of CasSD is 2.23. Friction ratio is an indicator of size and shape of a protein. Empirically, it has been shown that a nearly globular protein exhibits a friction ratio of around 1.2 to 1.3, whereas an elongated or branched protein has a ratio of 2.0 to 3.0 [44]. Accordingly, CasSD was thought to assume a non-globular and elongated shape, behaving closely to the previously defined native coil-like protein [45]. Combining these results, we can begin to formulate that CasSD is a coil-like intrinsically disordered monomeric 35 kDa protein that persists in a heterogeneous ensemble of predominantly elongated prolate forms. It has an expanded *R*_H_ compared to folded proteins of the same length but smaller than the calculated value for the chemically denatured form, which would have an *R*_H_ value of approximately 61 Å [46].

### CasSD exhibits a typical IDP Circular Dichroism spectrum

**Figure 6:**
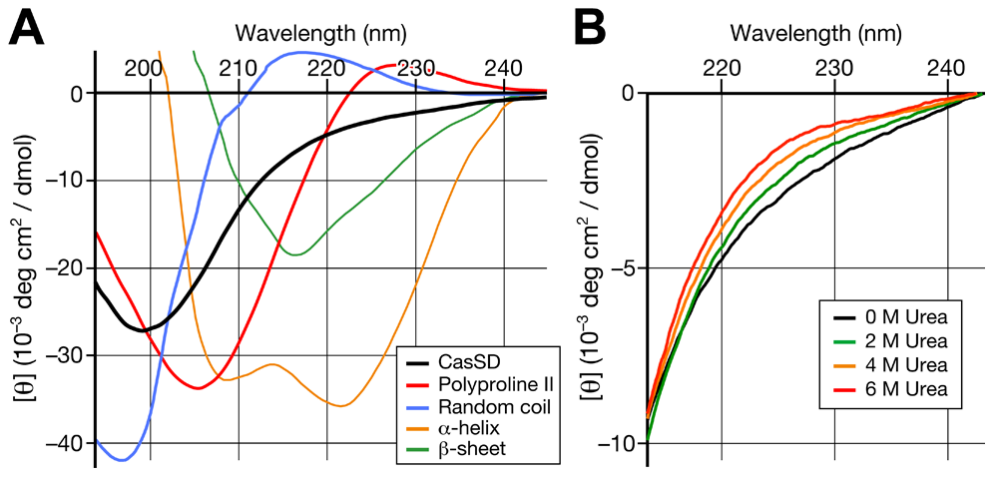
Circular dichroism (CD) spectra of CasSD. (A) CD spectrum of CasSD at 0.2 mg/mL concentration in 10 mM sodium phosphate pH 7.8 (black line). Standard spectra for reference samples with known secondary structures are also given for comparison. Thin orange: α-helix; Thin green: β-sheet; Red: polyproline II; Blue: random coil. (B) Increase of molar ellipticity in the 220–240 nm region of the CasSD CD spectrum with increasing concentration of urea.

The circular dichroism (CD) spectrum of CasSD was collected to determine what type of secondary structure is present. The result shows that CasSD lacks α-helices or β-sheets as its predominant secondary structure constituents (Figure 6A). Negative ellipticity at around 215 nm and the strong the negative peak at 200 nm suggests the presence of PPII-type dihedral angle conformations in residues including proline and other amino acids [47], [48]. An increasing concentration of urea, up to 6 M, does not effect a large change in the spectra, confirming lack of α-helices or β-sheets (Figure 6B). The CD spectrum of CasSD appears nearly identical to those of other intrinsically disordered or unstructured proteins including ActA [49], β-casein [50], bovine viral diarrhea virus core [51] and a synthetic hydrophilic recombinant gelatin [52]. Compositional bias varies in these four examples from 4.9–22.4% proline, 5.8–33.7% glycine and 3.0–20.6% lysine. The similarity in these CD spectra indicates that unstructured proteins exhibit similar subsets of backbone conformational space that are tolerant to a wide range of amino acid compositional biases. Slightly negative ellipticity in the 222 nm region has been interpreted in the past to possibly indicate the minor presence of α-helix or β-sheet secondary structure. However, a new interpretation arises from recent results from a comprehensive library of 400 blocked dipeptide CD spectra [29] which shows that this spectral feature at 222 nm is a general property of amino acid pairs in two dominant conformations, PPII and β, where the β conformers are not stabilized by strand-paired hydrogen bonds. The negative ellipticity feature at 222 nm in blocked dipeptide CD spectra is also temperature dependent as is the 222 nm feature of an IPD, ActA [49]. The dipeptide library results show that these full-length protein CD spectra are consistent with a population of dominant PPII and unpaired β conformations, with β conformations increasing with temperature. General decrease of the ellipticity at 222 nm over increasing temperature was in fact observed with CasSD (Figure S4A), and similar decrease of the ellipticity with increase in the buffer acidity was also observed with CasSD (Figure S4B). This observation follows precisely the known behavior of IDPs termed “turned out” response to heat and changes in pH [53]. This partial folding of IDPs under elevated temperature and low pH is thought to be induced by increased hydrophobic interaction and dampened electrostatic repulsion among the protein backbones, leading to the shift of the conformational states of CasSD toward β. In addition, there is a urea-induced increase of ellipticity in CasSD at around 222 nm (Figure 6B), which suggests that urea changes the conformational states of CasSD, possibly altering the mixed populations of PPII and β conformations towards β as suggested by recent NMR results [54]. Such shift of conformations toward β would enlarge the ensemble *R*_H_, as observed in the case of chemically denatured ActA by SEC [49].

### Sequence analysis of CasSD and its pseudo-repeats

**Figure 7:**
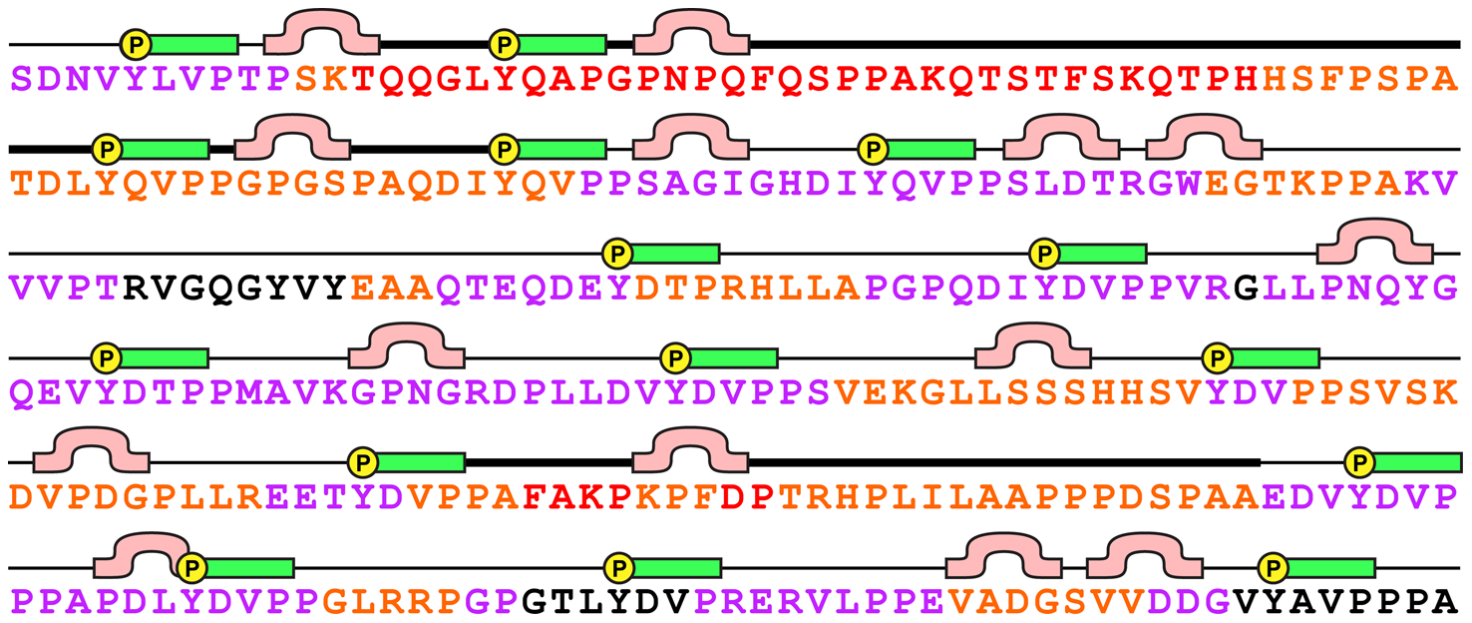
Consensus of predictions of CasSD disorderliness using multiple prediction programs. YxxP motif is represented by a green bar with a circled P representing the tyrosine residue to be phosphorylated. Residues predicted to be disordered by less than half of the programs are in black, and those predicted by progressively more programs are colored in purple, orange, and red. Lastly, a segment predicted by NetTurn P1.0 to assume a β-turn is shown by a pink Ω-shaped bar. See the Materials and Methods for details.

When the amino acid sequence of CasSD is analyzed using various disorder prediction programs listed in the Materials and Methods section, all programs indicate that the predominant portion of the protein is disordered. High propensity for disorder is predicted for residues 115–189 and 265–394 (Figure 7, thick-lined segments with orange and red letters), with highest probability predicted for residues 124–174 and 294–394 (red letters). On the other hand, the central and C-terminal regions (residues 190–264 and 386–410, respectively) are predicted to be least disordered (black letters) within the domain. In line with those disorder predictions, most sequence-based secondary structure prediction algorithms also assign CasSD to be comprised of random coil for its entire length, a typical result obtained for IDPs. NetTurn P1.0, a program for sequence-based prediction for occurrence of β-turn motifs [55], suggests that turn-prone positions (Figure 7, Ω-shaped pink bars) exist in between most of the YxxP motifs (Figure 7, yellow circled Ps with green bars). We note that to be stable turns, they would require flanking β-strands forming antiparallel hydrogen bonds, which is not supported by the CD data. Thus, we speculate that those predicted “β-turn motifs” may represent positions that introduce discontinuity into the CasSD structure. A multiple alignment of CasSD sequences from 11 placental mammalian species is shown in Figure S5. Those sequences have 71% identity across the domain, but the spacing of the YxxP motifs are absolutely conserved in all species. Occurrence of highly variable positions relative to the YxxP motifs is also conserved well and coincides with the turn-prone regions suggested in Figure 7. This highly conserved motif organization found among different CasSDs hints toward functional importance of the spatial arrangement of the YxxP motifs.

### TraDES structure ensemble analysis of CasSD

**Figure 8:**
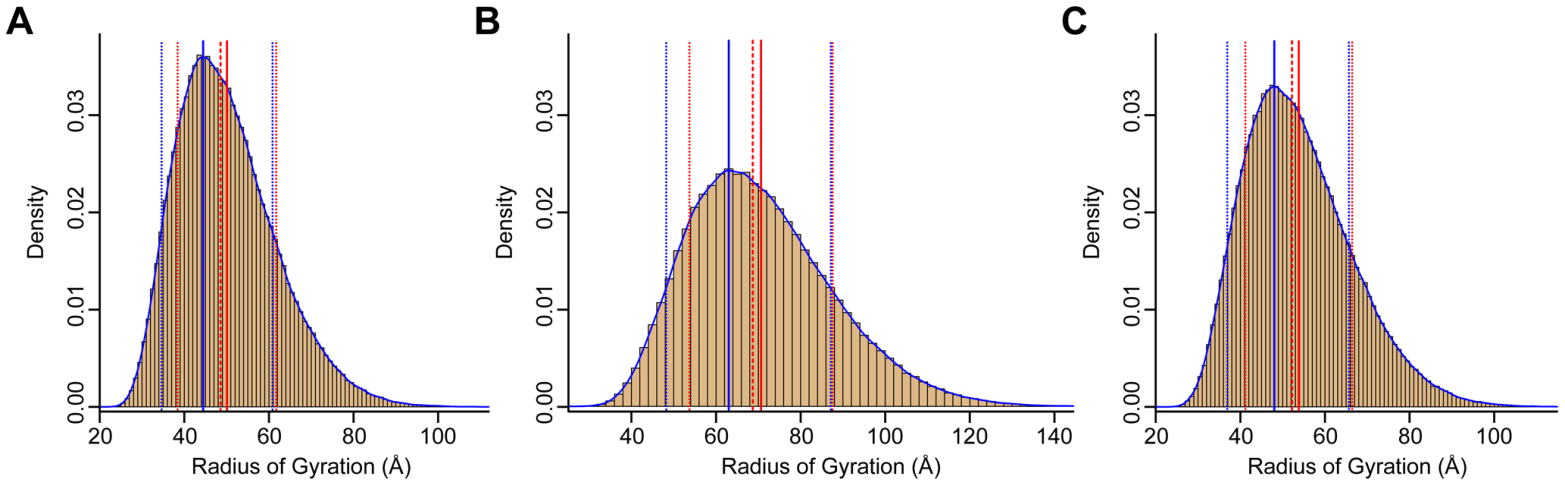
Histograms from TraDES sampling. (A) Coil-sampled ensemble. (B) β-sampled ensemble. (C) 3-state sampled ensemble. Solid blue line is the peak value. Solid red line is the mean value. Dashed red line is the median. Dotted red lines are at standard deviation, however the curves are not Gaussian, so the dotted blue lines bound the full-width at half max (FWHM).

**Figure 9:**
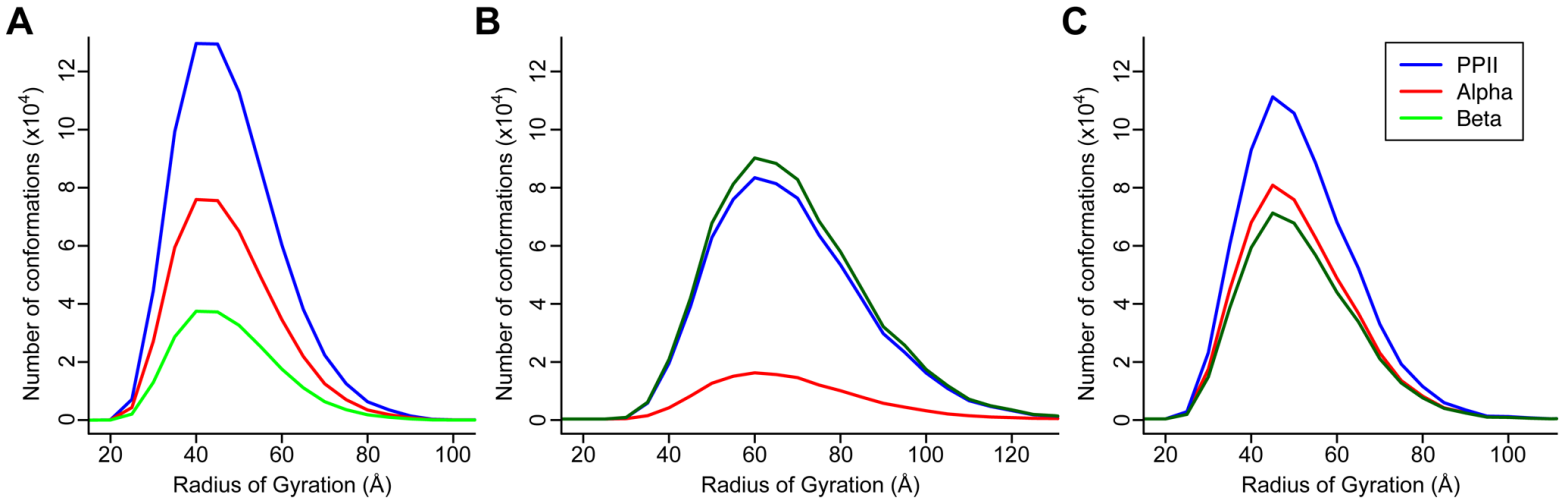
Relative abundance of sampled conformations. The relative frequency of PPII (blue line), α (red line) and β (green line) conformations in the (A) coil-sampled, (B) β-sampled and (C) 3-state ensembles. The frequencies are plotted against the radius of gyration that is binned into 5 Å windows.

The GOR [56] 3-state secondary structure prediction of CasSD is shown in Table S1, which represents the weights applied internally by the TraDES-2 package to the three basis sets of dictionary, α-, β- and coil subsets of φ,ψ dihedral angles, for the conformational sampling. TraDES samples Ramachandran space using these frequencies as a cumulative distribution function. The input dictionary ψ and φ distributions are obtained from non-secondary-structure regions of 7,030 representative non-redundant X-ray and NMR structures. There are significant differences in the three ensembles of backbone conformational space-sampling that are caused by the different sampling weights. The distribution of radius of gyration (*R*_Gyr_) values extracted from the three different simulations show distinct differences (Figures 8A, B and C). The mean *R*_Gyr_ value of coil-biased ensemble, 50.0 Å, matches the experimentally determined structure the best. The GOR 3-state biased conformations (mean *R*_Gyr_ of 53.8 Å) are similar to the coil-biased conformations as the predicted secondary structures were almost completely coil. The β-sampled conformations show a significantly enhanced average radius of 70.7 Å. A sampling of structures extracted from the three ensembles shows different proportions of PPII regions in the structures (Figure 9A, B and C). Clearly, the PPII regions are more abundant in the coil-sampled and the 3-state conformational ensembles, especially around the region of the experimentally measured value. However, it is only in the coil-sampled ensemble that we observe an enrichment of the PPII conformation at the expense of β structure. This is in good agreement with the results of the CD experiments. While the simulated structures do not provide accurate predictions at the level of single amino acid residues, it does provide a qualitative picture of the general behavior of protein conformational space. As the coil-biased conformations match the experimentally measured determined value of *R*_H_ the best and apparently reproduce the secondary structure content better than the other ensembles, it is reasonable to conclude that the average conformation sampled in this ensemble is a good approximation to the reality.

## Discussion

The structural and sequence properties of CasSD underlie an unknown stretch-based force detection mechanism. The experimental results obtained during the current study show that CasSD is devoid of α-helix and β-sheet structures and contains significant local PPII-type structure throughout its entire length. While it was earlier suggested that a stable compact structure of CasSD might hide tyrosine residues from phosphorylation that initiates downstream signaling events, our current results are inconsistent with this earlier hypothesis [10]. Instead, CasSD appears to contain conserved short blocks of sequence whose elongated structure is most likely comprised of local PPII-type left-handed helices on the C-terminal side of each tyrosine substrate (green bars next to yellow circled Ps in Figure 7). These short PPII blocks seem to be often flanked by sequence regions that are both variable in sequence and prone to form turn-like elements (Ω-shaped pink bars in Figure 7), possibly introducing structural breaks in the PPII-rich domain. We suspect that this closely interspersed structure–sequence organization prevents CasSD from forming regular secondary structure and packing tightly into a globular state. The computed *R*_Gyr_ and relative secondary structure content for the coil-sampled ensemble best reproduces the corresponding values deduced from the experimentally measured Stokes radii (*R*_H_) and CD spectra, respectively. The *R*_Gyr_ distribution of the coil-sampled ensemble, which has a fewer proportion of β structure, is also narrower (Figures 8A) with full width at half-maximum (FWHM) of 26.2 Å as compared to 38.9 Å (Figures 8B) and 28.8 Å (Figures 8C) for the β-sampled and 3-state ensembles, respectively. This can also be attributed to the increase in β-to-PPII ratio in the 3-state- and β-sampled ensembles. Thus, we conclude that the coil-sampled prolate state having low β and high PPII secondary structural content approximates the observed solution structure of CasSD, whereas the most elongated of the β structures likely approach the mechanically stretched forms of CasSD.

**Figure 10:**
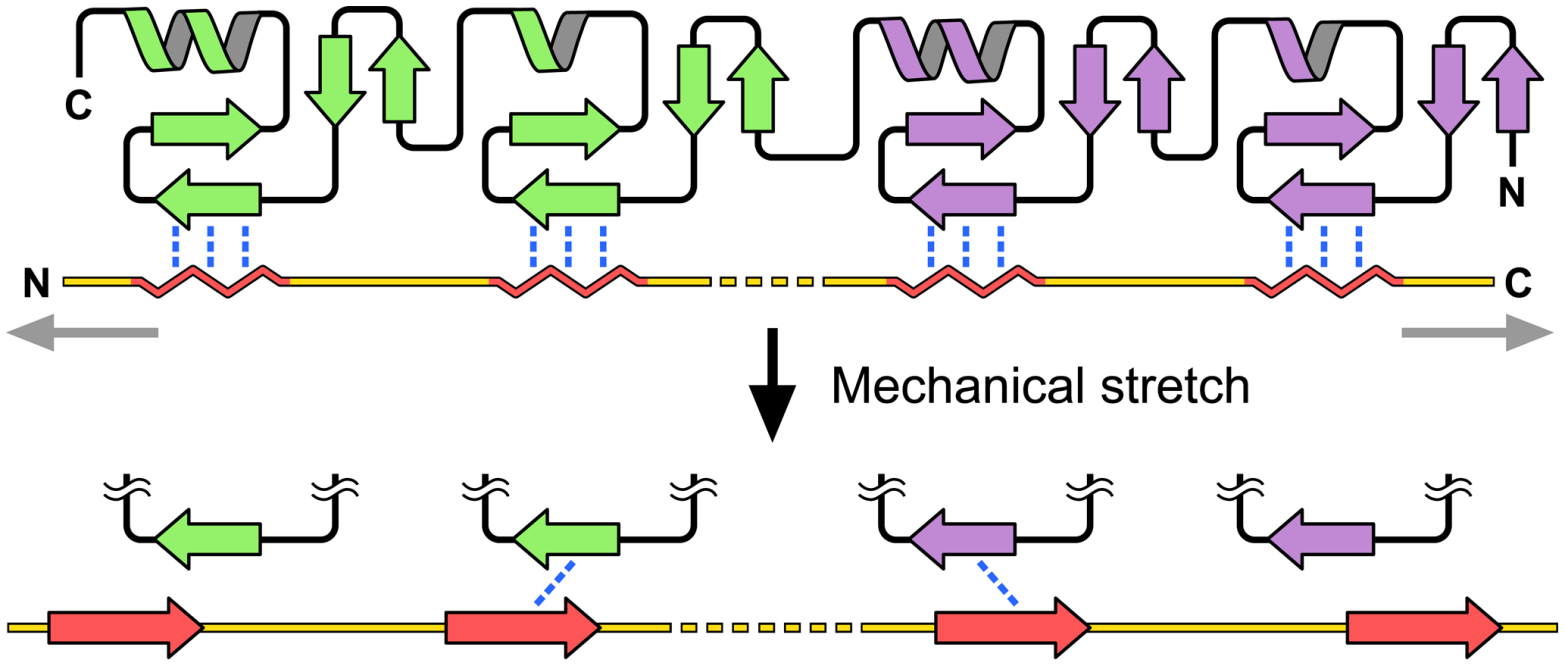
LIM pair binding peptides and spacer regions. Simple hypothetical model of a LIM domain pair (shown in green and purple ribbon diagram) bound to CasSD shown in yellow with its secondary structural elements colored in red. Wavy red lines and red arrows represent PPII helices and β-strands, respectively. Hydrogen bonds between LIM domains and CasSD are depicted in blue dotted lines. Mechanical stretching force is represented by gray arrows.

Results of our experimental and computational analyses suggest that stretching of CasSD is likely to elongate without resistance by undergoing a transformation from non-proline PPII and isolated α dihedral angle-based random coil structures into an elongated configuration with mixed β dihedral angles that appear wherever there are no local proline ring constraints. The conformational propensities of the CasSD ensemble do not appear sufficiently compact to maintain the overall ensemble in such a fashion that the YxxP phosphorylation motifs would be all simultaneously protected from phosphorylation by Src family kinases when the molecule is not stretched. The possibility arises that, instead, the unstretched CasSD may be blocked by several LIM domains present in LIM domain-containing proteins, such as zyxin and TRIP6 that are in fact known to bind p130Cas [11]. In addition, we note that zyxin itself shows stretch sensing properties [57], [58] where zyxin, upon stretching, reinforces actin stress fibers [58] and accumulates in the nucleus where it may be involved in gene regulation [57] as are other LIM domain-containing transcription factors [59], including homeodomain proteins [60]. If there is indeed a complex between p130Cas and zyxin or TRIP6 in the unstretched state of a focal adhesion, it can be speculated that the release of the LIM domains from p130Cas for phosphorylation of its substrate domain might be accomplished by physical stretching of such a complex. This raises the mechanistic question of how a mechanical force can disrupt a pre-existing LIM-domain–CasSD complex. Currently, very little is known about the relationship between stretch-sensing and gene expression despite its known clinical relevance in hypertension [61]. Our biophysical and computational experiments have clearly shown that there are significant PPII regions in CasSD. This ties up with the observation that CasSD is known to bind LIM domains. Analysis of LIM domain structures [62]–[64] reveals that LIM domains bind their substrate peptides in PPII conformation. This suggests that LIM domains likely bind to p130Cas at the PPII-rich CasSD. Any change to the backbone PPII conformation of CasSD, for instance by the application of a mechanical force that elongates the peptide and converts the PPII region to a β-stranded region, can lead to misalignment of hydrogen bonding partners between LIM domains and LIM-binding motifs in CasSD (Figure 10, moving from top to bottom panel). This would result in weakening of the LIM domain–CasSD interactions, allowing LIM domains to dissociate from CasSD and expose CasSD to Src family kinases for subsequent phosphorylation. Furthermore, LIM domain-containing proteins frequently carry two to three copies of LIM domain in tandem repeats. Thus, segments of LIM-binding substrate peptides that directly interact with LIM domains also occur in a relatively regular interval. As pointed out earlier (Figure S5), CasSDs across different mammalian species show an absolute conservation of the spacing of the YxxP motifs. This may be a reflection of the sensitivity of the stable LIM domain–CasSD complex formation toward spacing of the LIM-binding motifs present in CasSD that would allow formation of optimal hydrogen bonding and other favorable interactions between the two binding partners. Breaking of a single hydrogen bond requires a weak force that is approximately 5 pN in magnitude. We believe that the application of forces of this magnitude or slightly higher would rupture the hydrogen bonds between CasSD and the LIM domains bound to it. While we have no direct experimental evidence for such a mechanism currently, this model is proposed here to account for the good agreement observed in the experimental and computational analyses of the biological, biophysical and structural characteristics of CasSD.

## Materials and Methods

### Total internal reflection fluorescence microscopy and immunofluorescence analysis

p130Cas-deficient mouse embryonic fibroblasts expressing p130Cas tagged with GFP (GFP–p130Cas) were grown overnight in DMEM containing 10% FBS on a 50 µg/ml collagen-coated μ-Dish (ibidi, Martinsried, Germany) to form a monolayer. The cells were then treated with DMSO (0.1%) or 10 µM blebbistatin for 1 hour and scratched by a pipette tip 1.5 hours before fixation. This scratching of the cells simulates wounding of the monolayer. Cells were fixed with cold methanol for 20 minutes at −20°C, permeabilized with 0.1% Triton X-100 in PBS for 5 minutes at room temperature, blocked with 1% BSA in PBS for 1 hour at room temperature, incubated with a polyclonal antibody against phospho-p130Cas-Y165 (pCas-165) (Cell Signal Technology, Danvers, MA) as a primary antibody in PBS containing 1% BSA for overnight at 4°C, Lastly, the cells were incubated with an Alexa546-conjugated goat anti-rabbit IgG antibody (Invitrogen, Carlsbad, CA) as a secondary antibody for 1 hour at room temperature to fluorescently label pCas-165. Image acquisitions were performed on an IX81 inverted microscope (Olympus, Tokyo, Japan) equipped with an Olympus Total internal reflection fluorescence (TIRF) illumination arm, fiber-coupled 488 and 559 nm lasers to excite GFP and Alexa546, respectively, 60× 1.45 numerical aperture oil immersion objective lens, and an electron multiplying charge-coupled device camera with a 512-by-512 pixel chip (Evolve 512, Photometrics, Tucson, AZ).

### Immunoblotting analysis

1.5×10^6^ NIH3T3 cells were allowed to adhere to collagen-coated substrates overnight in DMEM containing 10% FBS. Subsequently, the cells were exposed to DMSO (0.1%), blebbistatin (50 µM), cytochalasin D (0.5 µM) or latrunculin B (0.5 µM) for 30 minutes, solubilized with SDS sample buffer, and subjected to SDS–PAGE. The gel was subjected to immunoblotting using anti-pCas-165 and anti-p130Cas (αCas3) antibody to visualize phospho-p130Cas and total p130Cas, respectively.

### Atomic force microscopy

Single-molecule stretching experiments were performed on a commercial AFM (DI Multimode AFM with Picoforce system, Veeco, Plainview, NY) in a buffer comprised of 25 mM HEPES and 125 mM sodium chloride at pH 7.4. CasSD–I27 (titin immunoglobulin domain 27)–CasSD–I27 was labeled with an N-terminal hexahistidine (His_6_)-tag for later binding to Ni-NTA-coated substrates. Before measurements, purified proteins were incubated on a Ni-NTA-coated slide [65] for 15 min. In AFM experiments, a gold-coated cantilever (HYDRA2R-100NGG, Appnano, Santa Clara, CA) with a spring constant around 15 pN/nm was repeatedly moved toward the slide surface 1 µm above, held at the surface with a contact force of 800 pN for 2 seconds, and then retracted from the surface at a constant velocity of 600 nm/s. When a single protein molecule [40] was absorbed to the cantilever, a force vs. extension curve was recorded. In the force-extension curves, each unfolding event was fitted by a worm-like-chain (WLC) model [40] to get the contour length. The difference in the contour length between consequent force peaks was treated as *ΔL* for the unfolding event associated with the former peak. Trajectories showing two unfolding force peaks of I27 domains (*ΔL* = 28±2 nm, *F*>100 pN) were chosen for final data processing, because any other (or none) feathers other than the two I27 peaks in such trajectories would come from CasSD.

### Cloning, expression, and purification of recombinant CasSD

Mouse CasSD was produced as a tobacco etch virus (TEV) protease-cleavable C-terminal His_6_-tagged protein in the *E. coli* BL21(DE3) Rosetta2 strain (Merck Biosciences, Darmstadt, Germany). Induction of the gene expression was achieved by 37°C incubation for three hours after addition of 400 µM isopropyl-β-d-thiogalactopyranoside (IPTG) to LB culture. Cell suspension in a lysis buffer (50 mM potassium phosphate pH 7.8, 300 mM potassium chloride, protease inhibitor cocktail VII (Merck Biosciences, Darmstadt, Germany) was sonicated and centrifuged to obtain a cleared cell lysate. This lysate was subjected to cobalt-affinity chromatography using HisPur cobalt resin (Thermo Scientific Pierce Protein Research Products, Rockford, IL). CasSD was eluted with 50 mM imidazole. The eluate was exchanged into a buffer composed of 10 mM potassium phosphate pH 7.5, 100 mM potassium chloride, 1 mM EDTA and 5% (v/v) glycerol using PD-10 desalting column (GE Healthcare, Waukesha, WI) and concentrated to approximately 1.5 mg/mL prior to being subjected to preparative SEC using a Superdex 10/300GL column on an ÅKTA purifier liquid chromatography system (GE Healthcare, Waukesha, WI). Purity of the protein was judged by SDS–PAGE. Rat CasSD was also produced as a N-terminal His_12_-tagged, C-terminal Avi-tagged protein using the *E. coli* BL21-CodonPlus (DE3)-RP strain (Agilent Technologies, Santa Clara, CA). Protein production was induced with 1 mM IPTG at 37°C for three hours in the M9 media supplemented with 3 µM thiamine. Cells were harvested and lysed in a denaturing lysis buffer containing 8 M urea. Cleared lysate was supplemented with sodium chloride to a final concentration of 50 mM before being subjected to nickel-affinity chromatography using Ni-NTA resin (QIAGEN, Hilden, Germany). Eluted rat CasSD was concentrated to approximately 1 mg/mL prior to being subjected to reversed-phase high-performance liquid chromatography using a semi-preparative Luna 10 micron C18(2) column (Phenomenex, Torrance, CA) on a Shimadzu LC-6AD semi-preparative system (Shimadzu Corporation, Kyoto, Japan). Samples were separated on a 0–80% acetonitrile linear gradient in water supplemented with 0.1% (v/v) trifluoroacetic acid. CasSD was eluted with 38–40% acetonitrile. The fractions containing CasSD were pooled and lyophilized. The lyophilized CasSD was kept at −80°C and used in subsequent experiments after reconstituting it in a suitable buffer.

### Analytical size exclusion chromatography

Purified mouse CasSD was subjected to analytical SEC using the same condition for preparative SEC described earlier. Purified CasSD was injected at 1.5 mg/mL concentration and eluted from the column at a flow rate of 0.5 mL/min in a buffer comprised of 10 mM potassium phosphate pH 7.5, 100 mM potassium chloride, 1 mM EDTA and 5% (v/v) glycerol. As a reference, proteins used as standard molecular weight references, namely horse spleen ferritin (type 1), bovine liver catalase, bovine serum albumin and bovine pancreatic ribonuclease A, were also subjected to gel-filtration chromatography using the same condition.

### Dynamic light scattering

DLS measurements were taken on DynaPro Titan (Wyatt Technology Corporation, Santa Barbara, CA) using the purified mouse CasSD. Measurements were collected at 1 to 3 mg/mL of purified CasSD in 10 mM potassium phosphate at pH 7.5, 100 mM potassium chloride, 1 mM EDTA and 5% glycerol at room temperature. Data analysis was performed using DYNAMICS V6 software to calculate the diffusion coefficient, hydrodynamic radius, molecular weight and polydispersity of CasSD.

### Sedimentation velocity analytical ultracentrifugation

Rat CasSD was subjected to SV-AUC experiment in 10 mM potassium phosphate at pH 7.5, 100 mM potassium chloride, 1 mM EDTA and 5% glycerol at a concentration of 1 mg/mL using XL-I analytical ultracentrifuge (Beckman-Coulter, Brea, CA). Samples were centrifuged at 40,000 rpm at 4°C over 7.3 hours with continuous scan from 5.8 to 7.2 cm at 0.003 cm interval. Data was fit using the program SednTerp (Alliance Protein Laboratories, Thousand Oaks, CA) and SedFit [66] with a continuous distribution model to obtain the experimental molecular weight, Stokes radius and frictional ratio of CasSD.

### Limited proteolysis

Limited proteolysis was performed on the purified mouse CasSD using trypsin as follows. A 2,000-fold excess of CasSD to protease was mixed in the reaction buffer (10 mM potassium phosphate and 20 mM calcium chloride at pH 7.8). Reaction was allowed to proceed on ice for 5, 10, 30, 60 and 120 minutes. At each time point, an aliquot is taken out and mixed with suitable protease inhibitor to quench the reaction. Aliquots were analyzed by SDS–PAGE.

### Circular dichroism spectroscopy

CD measurements are taken on JASCO J-715 spectropolarimeter (JASCO Corporation. Tokyo, Japan) using the purified mouse CasSD at 0.2 mg/mL concentration in 10 mM potassium phosphate at pH 7.8. Measurements were also taken on the rat CasSD in the presence of increasing concentrations of denaturing agents (0–6 M urea) in 10 mM potassium phosphate pH 7.8 at the same protein concentration to study the change in the secondary structure content of the protein upon denaturation. For the pH measurements, 10 mM potassium phosphate buffer was used for pH 6.6 and 7.5, while 100 mM citrate/phosphate buffer was used for pH 2.6, 3.6, 4.6 and 5.6.

### Sequence analysis

The mouse CasSD amino acid sequence isoform 1 (Accession NP_001185768.1) was subjected to disorderliness prediction by web-based algorithms DRIP-PRED [67], DISOPRED [68], IUPred [69], DisProt VL3H and VSL2P [70], Scratch [71], FoldUnfold [72], RONN [73], CSpritz [74], and FoldIndex [75]. Because each algorithm is based on a different theoretical framework, results were compared and combined to obtain a crude consensus of predicted disorderliness of this protein. Predicted degrees of disorderliness from different programs were normalized to a scale of 0 to 9 with 9 being the most disordered. Then, residues predicted as disordered by less than 50% of the programs were labeled as “less disordered (black),” 50–60% as “intermediately disordered (purple),” 70–80% as “disordered (orange),” and 90–100% as “extensively disordered (red).” The classification is arbitrary and hence only meant to illustrate a crude trend of the predicted disorderliness of the protein. Prediction for the occurrence of a β-turn was performed using the web-based program NetTurn P1.0 [55]. BLAST of the CasSD domain without SEG masking, using Blosum80 on the RefSeq database returned a number of vertebrate sequences, however only the subset of placental mammals showed conservation over the CasSD domain sequence. ClustalX [76] was used to illustrate the multiple sequence alignment of the 306-residue domain. Mouse CasSD composition included excessive proline residues (19.9%), yet the aligned sequences exhibited 71% sequence identity across the domain. A conserved deletion is observed in the common ancestor of horse and cow, corresponding to exactly one pseudo-repeat unit.

### Structure ensemble simulation for estimating the radius of gyration

As IDPs are not known to conform to any given 3D shape, an ensemble of possible representations of the 3D-shapes of the mouse CasSD sequence was generated. The new TraDES-2 *seq2trj* program available at http://trades.blueprint.org was used for this purpose [33]. The working of the TraDES software has been described in detail elsewhere [32], [33]. Briefly, given the secondary structure preferences of amino acids of a sequence, an ensemble of non-clashing 3D structures of the sequence is generated by assigning backbone Ramachandran angles (φ,ψ) according to the predicted (or assigned) secondary structure. In the new version of the TraDES-2 *seq2trj* program, the (φ,ψ) frequency information was derived from an updated non-redundant set of 7,030 structures including NMR single model structures for which no corresponding X-ray structure is available. The structures and chains used are listed in the TraDES-2 package data file *filtmmdblist*. The output of *seq2trj* is a sampling trajectory file containing sequence-weighted frequency φ,ψ with a 400×400 Ramachandran grid square resolution, representing the propensity for backbone conformational space that can be explored at each step in chain construction. Three sets of 300,000 structures each were constructed using the following biases to the φ,ψ-sampling frequencies:

- The GOR [56] algorithm was used to assign 3-state predicted secondary structure value to the sequence of mouse CasSD. Note that we verified these secondary assignments by also using PSIPRED [77] and found the predictions to be similar (Table S1). In this study the assignments of secondary structure were taken only from the GOR prediction. The φ,ψ-sampling frequencies were taken according to the predicted percentage of α, β and coil at each residue.
- The second set of 300,000 structures was sampled by weighting the φ,ψ frequencies to 100% coil conformations. The coil weighting effectively removes frequencies of φ,ψ angles found in detected α and β secondary structures and up-weights sampling from PPII conformations. Note that α and β φ,ψ angle instances remain present in the sampling frequencies from individual PDB residues adopting these dihedral angles in loops or coils outside of ordered secondary structure elements. The program also randomly chooses *cis*-proline conformers, which appear between 0–6 times for each structure, as previously described.
- The third ensemble of 300,000 was made by weighting the φ,ψ frequencies to 100% β conformations.

### Computing the radius of gyration and hydrodynamic radius

In generating an ensemble of 3D structures, TraDES computes the values of the following parameters for each structure: the radius of gyration (*R*_Gyr_), hydrodynamic radius, N-to-C-terminal distance, accessible surface area, hydrophobic accessible surface area, secondary structure content, and three statistical energy scoring functions. Of these, this study only concerns itself with the values of *R*_Gyr_. These values are computed during structure generation and are output in log files. 30,000 (10%) structures were randomly chosen from each of the 3 sampled sets of 300,000 structures. *R*_Gyr_ values of these samples were computed. The sampled structures are available at http://www.iiserpune.ac.in/~madhusudhan/pCas130_mechanosensing in VAL format. Each of the VAL format files could be converted to PDB format using the *str2pdb* package of the TraDES software. Input instructions are also provided to reproduce similar ensembles.

To compare the radius of gyration of sampled CasSD to experimental hydrodynamic radius, 17 additional tag residues with MG at the N-terminus and ENLYFQSLEHHHHHH at the C-terminus had to be accounted for. At this size range the Flory polymer ratio term corresponding to *R*_Gyr_^2^/*Nl* ^2^ (length *l* = 3.81 Å) has a constant distribution with peak to mean values in the range of 0.407–0.595. From this the *R*_Gyr_ correction for the additional *N* = 17 residues can be calculated to contribute an additional 1.3 (+/−0.1) Å to the peak, median or mean values. *R*_H_ as measured experimentally and the computed *R*_Gyr_ parameter are related [46] by the approximate *R*_Gyr_/*R*_H_ ratio of 1.06, based on measurements of urea denatured proteins. Estimates of urea-denatured protein *R*_Gyr_ estimates may be computed from protein length by the relation 1.927*N* ^0.598^ [78], which yields 61.0 Å for the tagged CasSD length of *N* = 306+17.

## Supporting Information

Figure S1 **Purified C-terminal His_6_-tagged p130Cas substrate domain (CasSD).** (A) Coomassie-stained SDS-PAGE gel of the purified sample of mouse CasSD. 1: CasSD; MW: molecular weight marker. Numbers correspond to the molecular weight in kDa of the bands to the right. (B) Molecular weight determination of the purified recombinant mouse CasSD by MALDI–TOF.
(TIF)

Figure S2 **Limited proteolysis of mouse CasSD.** Purified CasSD was treated with 1/2000 (w/w) amount of trypsin on ice for 0, 5, 30, 60 and 120 min. After quenching the reaction, the aliquots were analyzed by SDS–PAGE.
(TIF)

Figure S3 **Comparison of the behaviors of purified recombinant mouse and rat CasSD.** Purified recombinant mouse and rat CasSD were analyzed by (A) SDS–PAGE (1: CasSD; MW: molecular weight marker), (B) size exclusion chromatography and (C) circular dichroism. These results confirmed that the mouse and rat CasSDs behave in a virtually identical fashion in solution.
(TIF)

Figure S4 **Circular dichroism measurement of rat CasSD under varying temperatures and pH conditions.** Far UV circular dichroism measurements were taken on rat CasSD under (A) different temperature and (B) different pH, and change in the ellipticity measurements were examined at and around 222 nm.
(TIF)

Figure S5 **Multiple alignment of CasSD sequence domain fragments.** *Mus musculus* (house mouse) NP_001185768.1|gi|311771530/115–420, *Rattus norvegicus* (Norway rat) NP_037063.1 |gi|6978709/209–514, *Cricetulus griseus* (Chinese hamster) XP_003510213.1|gi|354496197/115–420, *Callithrix jacchus* (white-tufted-ear marmoset) XP_002761197.1|gi|296231589/115–420, *Canis lupus familiaris* (dog) XP_004437163.1|gi|478525953/108–413, *Ailuropoda melanoleuca* (panda) XP_002927764.1|gi|301784705/159–464, *Homo sapiens* (human) NP_001164185.1|gi|282398112/157–462, *Pan troglodytes* (chimpanzee) XP_003315268.1|gi|332846521/157–462, *Loxodonta africana* (African savanna elephant) XP_003417214.1|gi|344290979/118–423, *Equus caballus* (horse) XP_001916294.2|gi|338723073/120–412, *Bos taurus* (cattle) NP_001193627.1|gi|331999983/100–392.
(TIF)

Table S1 **CasSD 3-state secondary structure prediction using the GOR and PSIPRED methods.** Pred and Conf stand for prediction and confidence, respectively.
(DOCX)
