# Supplementary figures and images for: Biophysical Properties of Intrinsically Disordered p130Cas Substrate Domain — Implication in Mechanosensing
Source: PLoS Comput Biol. 2014 Apr 10;10(4):e1003532. doi: 10.1371/journal.pcbi.1003532 (PMC3983058; doi:10.1371/journal.pcbi.1003532)

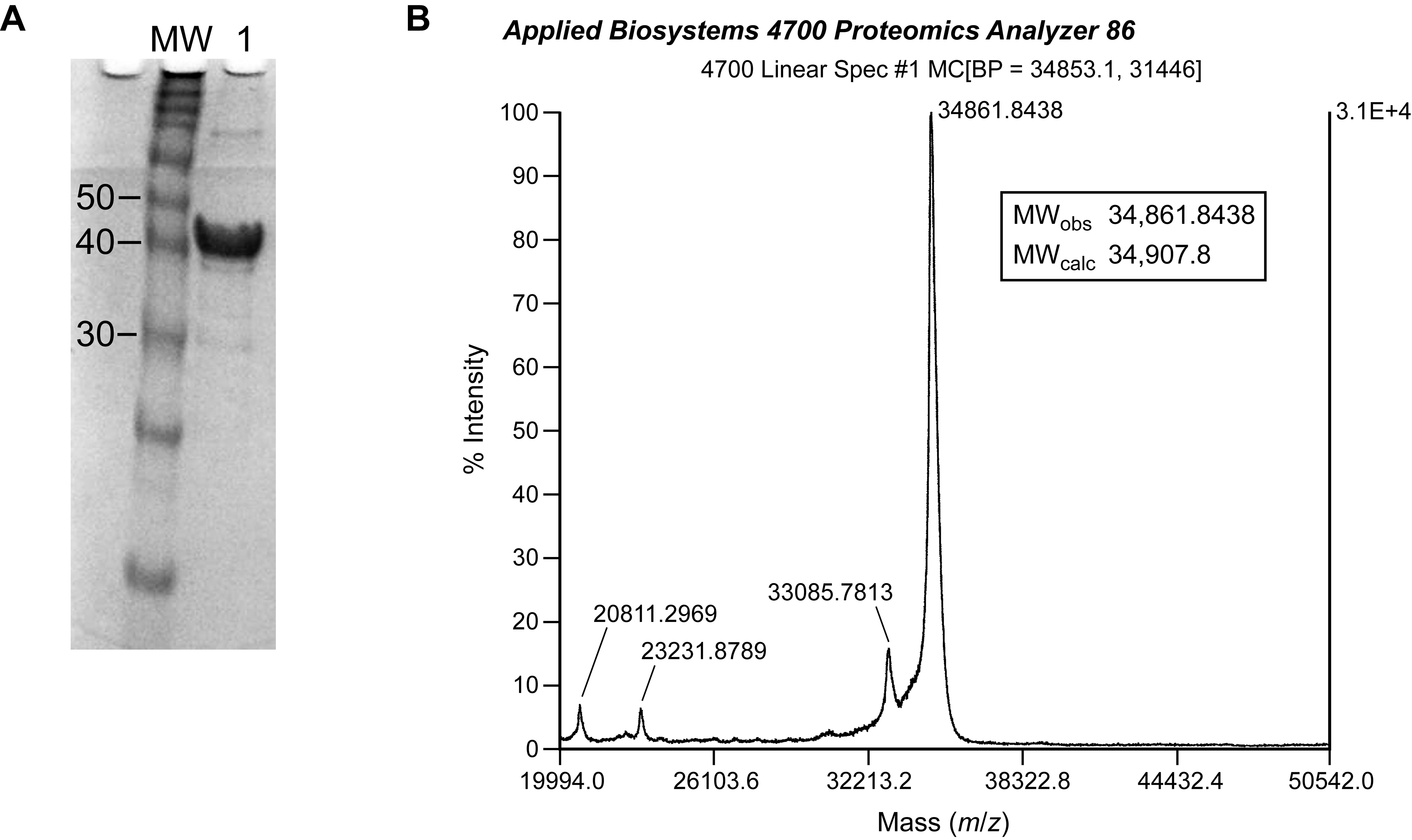

Supplement: Figure S1 — Purified C-terminal His6-tagged p130Cas substrate domain (CasSD). (A) Coomassie-stained SDS-PAGE gel of the purified sample of mouse CasSD. 1: CasSD; MW: molecular weight marker. Numbers correspond to the molecular weight in kDa of the bands to the right. (B) Molecular weight determination of the purified recombinant mouse CasSD by MALDI–TOF. (TIF) [file pcbi.1003532.s001.tif]

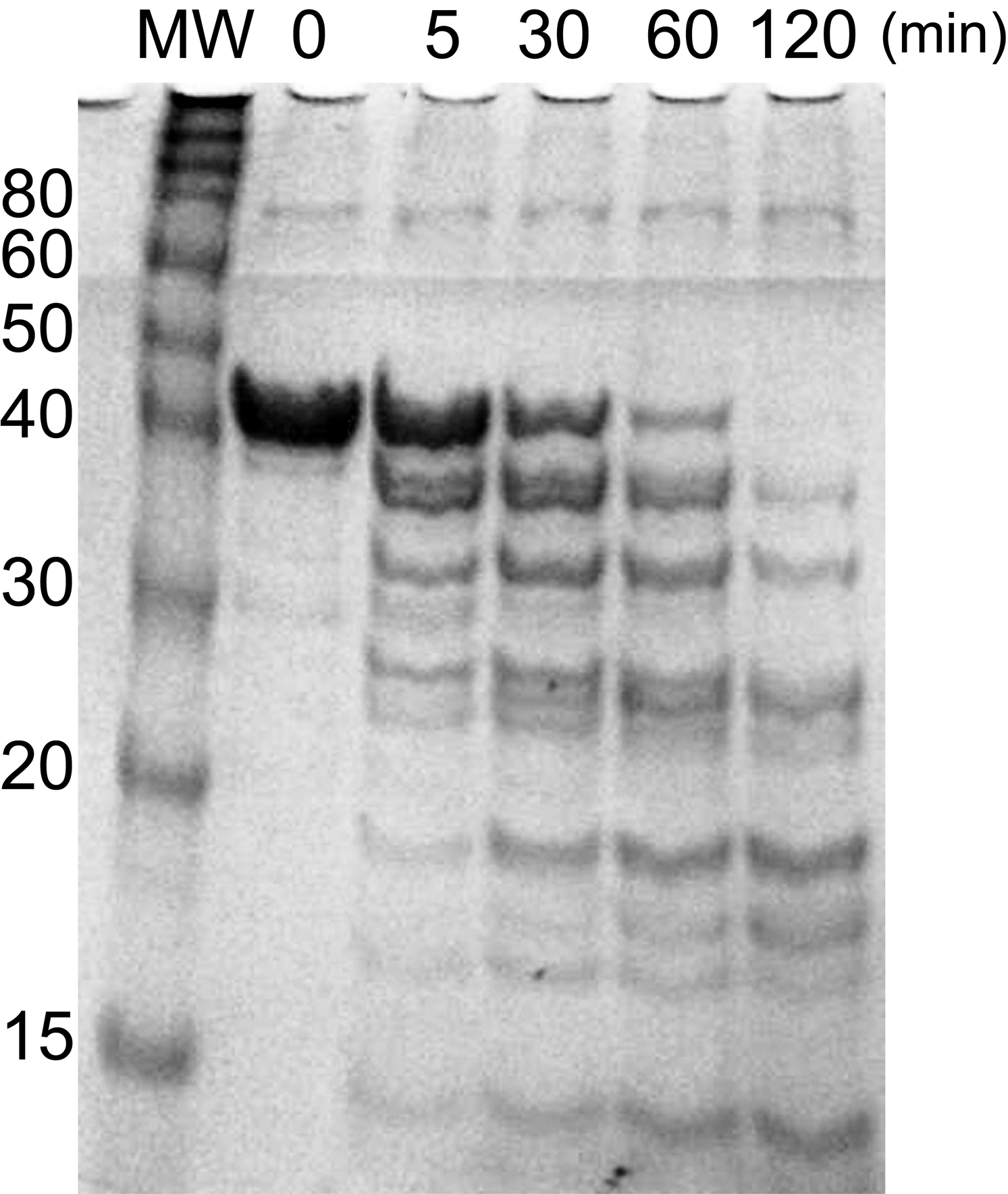

Supplement: Figure S2 — Limited proteolysis of mouse CasSD. Purified CasSD was treated with 1/2000 (w/w) amount of trypsin on ice for 0, 5, 30, 60 and 120 min. After quenching the reaction, the aliquots were analyzed by SDS–PAGE. (TIF) [file pcbi.1003532.s002.tif]

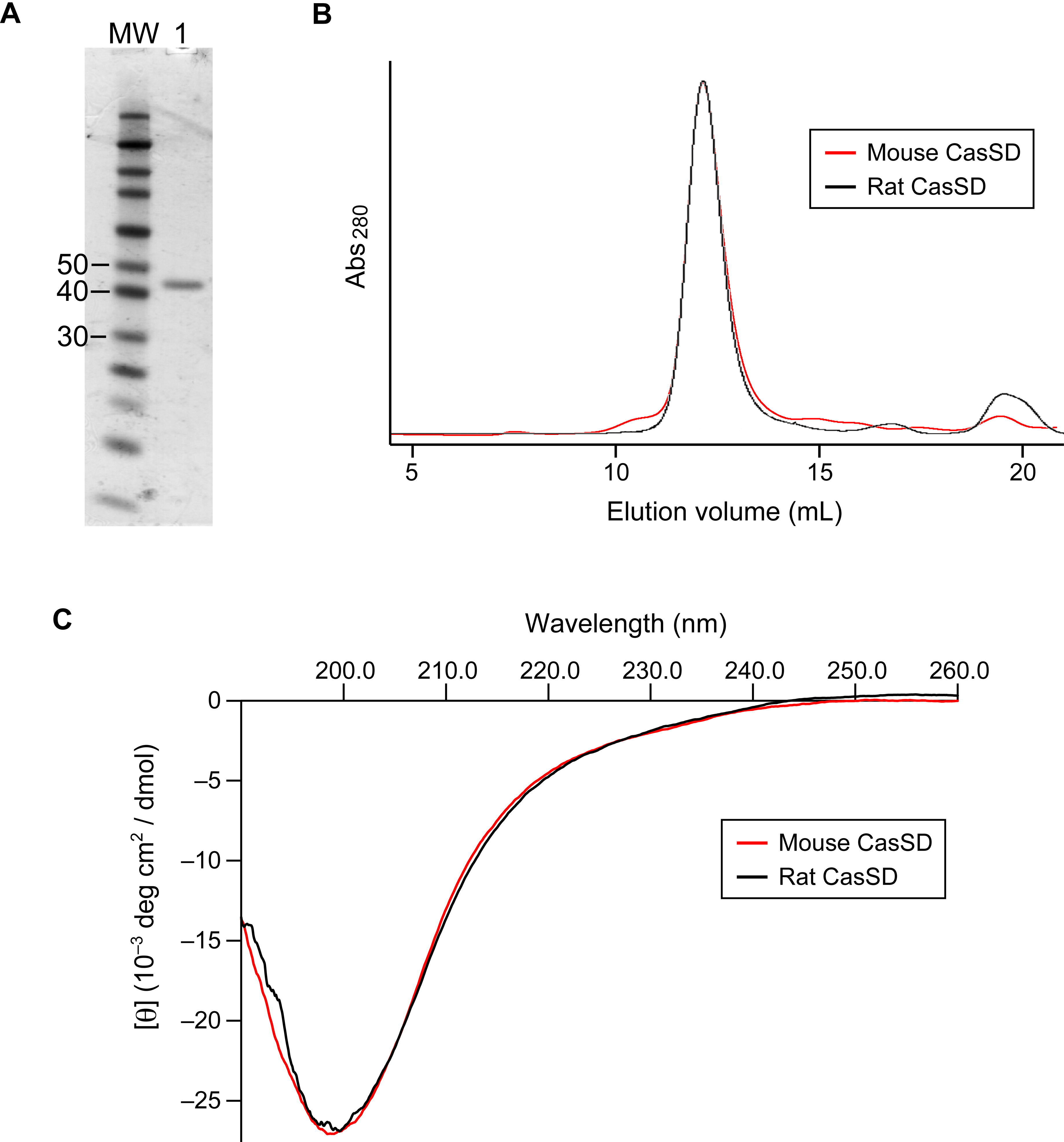

Supplement: Figure S3 — Comparison of the behaviors of purified recombinant mouse and rat CasSD. Purified recombinant mouse and rat CasSD were analyzed by (A) SDS–PAGE (1: CasSD; MW: molecular weight marker), (B) size exclusion chromatography and (C) circular dichroism. These results confirmed that the mouse and rat CasSDs behave in a virtually identical fashion in solution. (TIF) [file pcbi.1003532.s003.tif]

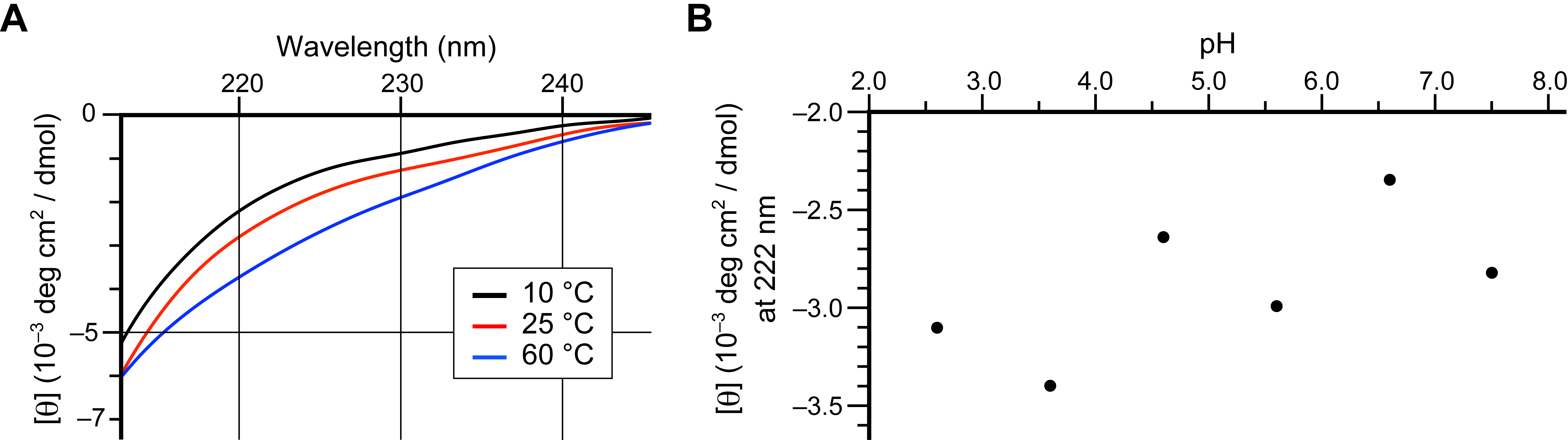

Supplement: Figure S4 — Circular dichroism measurement of rat CasSD under varying temperatures and pH conditions. Far UV circular dichroism measurements were taken on rat CasSD under (A) different temperature and (B) different pH, and change in the ellipticity measurements were examined at and around 222 nm. (TIF) [file pcbi.1003532.s004.tif]

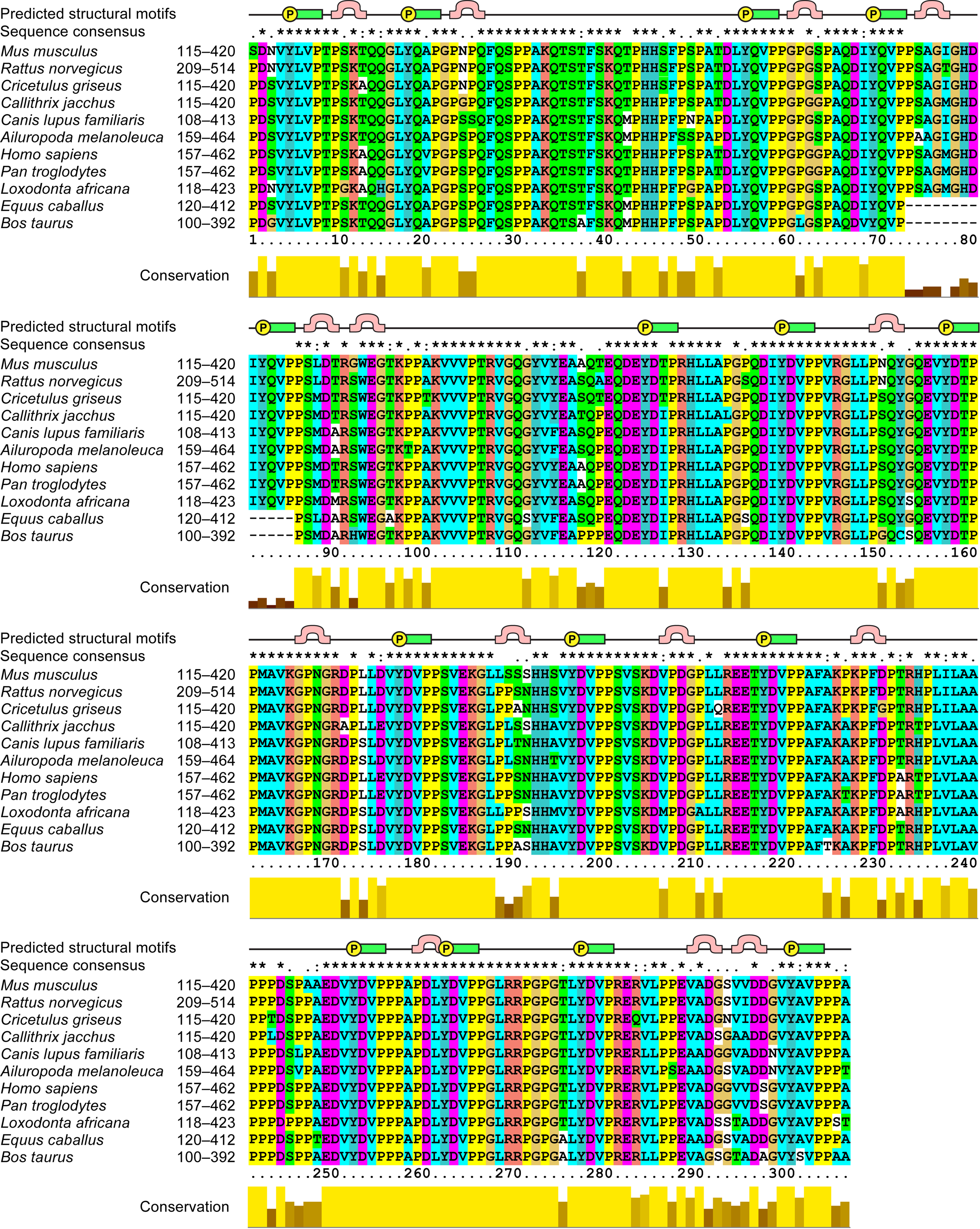

Supplement: Figure S5 — Multiple alignment of CasSD sequence domain fragments. Mus musculus (house mouse) NP_001185768.1|gi|311771530/115–420, Rattus norvegicus (Norway rat) NP_037063.1 |gi|6978709/209–514, Cricetulus griseus (Chinese hamster) XP_003510213.1|gi|354496197/115–420, Callithrix jacchus (white-tufted-ear marmoset) XP_002761197.1|gi|296231589/115–420, Canis lupus familiaris (dog) XP_004437163.1|gi|478525953/108–413, Ailuropoda melanoleuca (panda) XP_002927764.1|gi|301784705/159–464, Homo sapiens (human) NP_001164185.1|gi|282398112/157–462, Pan troglodytes (chimpanzee) XP_003315268.1|gi|332846521/157–462, Loxodonta africana (African savanna elephant) XP_003417214.1|gi|344290979/118–423, Equus caballus (horse) XP_001916294.2|gi|338723073/120–412, Bos taurus (cattle) NP_001193627.1|gi|331999983/100–392. (TIF) [file pcbi.1003532.s005.tif]
