# Supplementary material for: Biophysical Properties of Intrinsically Disordered p130Cas Substrate Domain — Implication in Mechanosensing
Source: PLoS Comput Biol. 2014 Apr 10;10(4):e1003532. doi: 10.1371/journal.pcbi.1003532 (PMC3983058; doi:10.1371/journal.pcbi.1003532)
Supplement: Table S1 — CasSD 3-state secondary structure prediction using the GOR and PSIPRED methods. Pred and Conf stand for prediction and confidence, respectively. (DOCX) [file pcbi.1003532.s006.docx]

| Sequence | |  | GOR RESULTS | | | |  | PSI-PRED RESULTS | |
| --- | --- | --- | --- | --- | --- | --- | --- | --- | --- |
|  |  |  | Pred. | H | E | C |  | Pred | Conf |
| 115 | S |  | C | 0 | 0 | 100 |  | C | 9 |
| 116 | D |  | C | 0 | 0 | 100 |  | C | 9 |
| 117 | N |  | C | 0 | 0 | 100 |  | C | 7 |
| 118 | V |  | C | 0 | 0 | 100 |  | C | 3 |
| 119 | Y |  | C | 0 | 2 | 98 |  | C | 4 |
| 120 | L |  | C | 0 | 8 | 92 |  | C | 3 |
| 121 | V |  | C | 0 | 18 | 82 |  | C | 4 |
| 122 | P |  | C | 0 | 27 | 73 |  | C | 9 |
| 123 | T |  | C | 2 | 29 | 69 |  | C | 9 |
| 124 | P |  | C | 4 | 16 | 80 |  | C | 9 |
| 125 | S |  | C | 5 | 11 | 85 |  | C | 9 |
| 126 | K |  | C | 13 | 10 | 77 |  | C | 9 |
| 127 | T |  | C | 17 | 15 | 68 |  | C | 9 |
| 128 | Q |  | C | 29 | 26 | 46 |  | C | 9 |
| 129 | Q |  | C | 22 | 38 | 40 |  | C | 9 |
| 130 | G |  | E | 17 | 51 | 32 |  | C | 8 |
| 131 | L |  | E | 12 | 57 | 31 |  | C | 3 |
| 132 | Y |  | E | 8 | 54 | 38 |  | C | 3 |
| 133 | Q |  | C | 5 | 45 | 50 |  | C | 5 |
| 134 | A |  | C | 3 | 27 | 71 |  | C | 8 |
| 135 | P |  | C | 0 | 11 | 89 |  | C | 9 |
| 136 | G |  | C | 0 | 6 | 93 |  | C | 9 |
| 137 | P |  | C | 0 | 7 | 93 |  | C | 9 |
| 138 | N |  | C | 0 | 8 | 92 |  | C | 9 |
| 139 | P |  | C | 0 | 9 | 90 |  | C | 9 |
| 140 | Q |  | C | 1 | 17 | 83 |  | C | 9 |
| 141 | F |  | C | 1 | 20 | 79 |  | C | 9 |
| 142 | Q |  | C | 1 | 20 | 79 |  | C | 8 |
| 143 | S |  | C | 1 | 17 | 82 |  | C | 9 |
| 144 | P |  | C | 1 | 11 | 87 |  | C | 9 |
| 145 | P |  | C | 3 | 9 | 88 |  | C | 9 |
| 146 | A |  | C | 6 | 12 | 82 |  | C | 8 |
| 147 | K |  | C | 10 | 13 | 77 |  | C | 8 |
| 148 | Q |  | C | 12 | 23 | 65 |  | C | 7 |
| 149 | T |  | C | 13 | 35 | 52 |  | C | 7 |
| 150 | S |  | E | 14 | 47 | 39 |  | C | 7 |
| 151 | T |  | E | 14 | 49 | 38 |  | C | 8 |
| 152 | F |  | E | 11 | 49 | 40 |  | C | 8 |
| 153 | S |  | E | 9 | 48 | 43 |  | C | 8 |
| 154 | K |  | C | 7 | 46 | 47 |  | C | 8 |
| 155 | Q |  | C | 4 | 46 | 50 |  | C | 9 |
| 156 | T |  | C | 2 | 43 | 55 |  | C | 8 |
| 157 | P |  | C | 1 | 35 | 64 |  | C | 8 |
| 158 | H |  | C | 1 | 38 | 61 |  | C | 9 |
| 159 | H |  | C | 1 | 37 | 62 |  | C | 8 |
| 160 | S |  | C | 1 | 25 | 74 |  | C | 8 |
| 161 | F |  | C | 1 | 25 | 74 |  | C | 9 |
| 162 | P |  | C | 1 | 20 | 79 |  | C | 9 |
| 163 | S |  | C | 2 | 14 | 84 |  | C | 9 |
| 164 | P |  | C | 3 | 13 | 84 |  | C | 9 |
| 165 | A |  | C | 4 | 17 | 79 |  | C | 9 |
| 166 | T |  | C | 5 | 27 | 68 |  | C | 9 |
| 167 | D |  | C | 4 | 35 | 61 |  | C | 8 |
| 168 | L |  | E | 4 | 55 | 41 |  | C | 6 |
| 169 | Y |  | E | 2 | 58 | 41 |  | C | 3 |
| 170 | Q |  | E | 1 | 55 | 44 |  | C | 3 |
| 171 | V |  | C | 1 | 42 | 58 |  | C | 7 |
| 172 | P |  | C | 0 | 20 | 80 |  | C | 9 |
| 173 | P |  | C | 0 | 10 | 90 |  | C | 9 |
| 174 | G |  | C | 0 | 6 | 94 |  | C | 9 |
| 175 | P |  | C | 0 | 5 | 95 |  | C | 9 |
| 176 | G |  | C | 0 | 7 | 93 |  | C | 9 |
| 177 | S |  | C | 1 | 8 | 91 |  | C | 9 |
| 178 | P |  | C | 2 | 13 | 86 |  | C | 9 |
| 179 | A |  | C | 3 | 21 | 76 |  | C | 9 |
| 180 | Q |  | C | 4 | 35 | 61 |  | C | 9 |
| 181 | D |  | E | 7 | 50 | 44 |  | C | 8 |
| 182 | I |  | E | 7 | 65 | 27 |  | C | 5 |
| 183 | Y |  | E | 7 | 61 | 32 |  | C | 0 |
| 184 | Q |  | E | 4 | 63 | 33 |  | C | 0 |
| 185 | V |  | E | 4 | 49 | 47 |  | C | 4 |
| 186 | P |  | C | 2 | 34 | 64 |  | C | 9 |
| 187 | P |  | C | 3 | 20 | 77 |  | C | 9 |
| 188 | S |  | C | 3 | 18 | 79 |  | C | 9 |
| 189 | A |  | C | 6 | 24 | 70 |  | C | 9 |
| 190 | G |  | C | 4 | 28 | 68 |  | C | 9 |
| 191 | I |  | C | 4 | 41 | 55 |  | C | 9 |
| 192 | G |  | E | 3 | 51 | 47 |  | C | 9 |
| 193 | H |  | E | 2 | 63 | 35 |  | C | 9 |
| 194 | D |  | E | 3 | 67 | 30 |  | C | 7 |
| 195 | I |  | E | 4 | 71 | 25 |  | C | 3 |
| 196 | Y |  | E | 3 | 68 | 30 |  | C | 2 |
| 197 | Q |  | E | 1 | 57 | 41 |  | C | 3 |
| 198 | V |  | E | 2 | 52 | 47 |  | C | 7 |
| 199 | P |  | C | 1 | 28 | 71 |  | C | 9 |
| 200 | P |  | C | 3 | 21 | 77 |  | C | 9 |
| 201 | S |  | C | 4 | 18 | 78 |  | C | 8 |
| 202 | L |  | C | 6 | 25 | 68 |  | C | 9 |
| 203 | D |  | C | 8 | 25 | 67 |  | C | 9 |
| 204 | T |  | C | 10 | 35 | 55 |  | C | 8 |
| 205 | R |  | C | 9 | 35 | 56 |  | C | 9 |
| 206 | G |  | C | 5 | 33 | 62 |  | C | 9 |
| 207 | W |  | C | 7 | 36 | 57 |  | C | 8 |
| 208 | E |  | C | 6 | 32 | 63 |  | C | 7 |
| 209 | G |  | C | 2 | 28 | 69 |  | C | 9 |
| 210 | T |  | C | 1 | 20 | 79 |  | C | 8 |
| 211 | K |  | C | 1 | 20 | 79 |  | C | 9 |
| 212 | P |  | C | 1 | 18 | 81 |  | C | 9 |
| 213 | P |  | C | 1 | 22 | 77 |  | C | 9 |
| 214 | A |  | C | 2 | 41 | 57 |  | C | 9 |
| 215 | K |  | E | 2 | 66 | 32 |  | C | 5 |
| 216 | V |  | E | 2 | 80 | 18 |  | C | 2 |
| 217 | V |  | E | 2 | 86 | 12 |  | C | 5 |
| 218 | V |  | E | 2 | 84 | 14 |  | C | 6 |
| 219 | P |  | E | 3 | 79 | 17 |  | C | 8 |
| 220 | T |  | E | 5 | 74 | 22 |  | C | 7 |
| 221 | R |  | E | 8 | 65 | 27 |  | C | 5 |
| 222 | V |  | E | 10 | 56 | 34 |  | C | 6 |
| 223 | G |  | E | 8 | 51 | 41 |  | C | 8 |
| 224 | Q |  | E | 16 | 45 | 39 |  | C | 8 |
| 225 | G |  | E | 16 | 52 | 32 |  | C | 7 |
| 226 | Y |  | E | 24 | 52 | 24 |  | C | 5 |
| 227 | V |  | E | 36 | 46 | 18 |  | C | 5 |
| 228 | Y |  | E | 40 | 43 | 17 |  | C | 7 |
| 229 | E |  | H | 50 | 31 | 19 |  | C | 8 |
| 230 | A |  | H | 56 | 24 | 20 |  | C | 8 |
| 231 | A |  | H | 55 | 19 | 26 |  | C | 9 |
| 232 | Q |  | H | 51 | 18 | 31 |  | C | 8 |
| 233 | T |  | H | 42 | 19 | 39 |  | C | 6 |
| 234 | E |  | C | 42 | 12 | 46 |  | C | 6 |
| 235 | Q |  | C | 39 | 11 | 50 |  | C | 6 |
| 236 | D |  | C | 41 | 9 | 50 |  | C | 7 |
| 237 | E |  | C | 47 | 5 | 48 |  | C | 7 |
| 238 | Y |  | C | 38 | 7 | 55 |  | C | 6 |
| 239 | D |  | C | 22 | 12 | 66 |  | C | 7 |
| 240 | T |  | C | 21 | 19 | 59 |  | C | 9 |
| 241 | P |  | C | 21 | 25 | 54 |  | C | 9 |
| 242 | R |  | C | 23 | 30 | 47 |  | C | 9 |
| 243 | H |  | C | 16 | 36 | 48 |  | C | 9 |
| 244 | L |  | C | 11 | 33 | 56 |  | C | 9 |
| 245 | L |  | C | 8 | 30 | 62 |  | C | 9 |
| 246 | A |  | C | 6 | 20 | 74 |  | C | 9 |
| 247 | P |  | C | 3 | 12 | 86 |  | C | 9 |
| 248 | G |  | C | 1 | 9 | 89 |  | C | 9 |
| 249 | P |  | C | 1 | 11 | 88 |  | C | 9 |
| 250 | Q |  | C | 1 | 16 | 83 |  | C | 9 |
| 251 | D |  | C | 2 | 24 | 74 |  | C | 9 |
| 252 | I |  | C | 2 | 44 | 53 |  | C | 7 |
| 253 | Y |  | C | 2 | 41 | 57 |  | C | 5 |
| 254 | D |  | C | 2 | 38 | 60 |  | C | 5 |
| 255 | V |  | C | 3 | 41 | 56 |  | C | 8 |
| 256 | P |  | C | 3 | 36 | 61 |  | C | 9 |
| 257 | P |  | C | 3 | 33 | 64 |  | C | 9 |
| 258 | V |  | C | 4 | 32 | 64 |  | C | 8 |
| 259 | R |  | C | 5 | 45 | 50 |  | C | 9 |
| 260 | G |  | C | 3 | 45 | 52 |  | C | 8 |
| 261 | L |  | C | 5 | 40 | 55 |  | C | 9 |
| 262 | L |  | C | 6 | 32 | 62 |  | C | 9 |
| 263 | P |  | C | 8 | 26 | 66 |  | C | 9 |
| 264 | N |  | C | 9 | 26 | 65 |  | C | 9 |
| 265 | Q |  | C | 10 | 24 | 66 |  | C | 8 |
| 266 | Y |  | C | 9 | 26 | 65 |  | C | 9 |
| 267 | G |  | C | 6 | 36 | 58 |  | C | 9 |
| 268 | Q |  | C | 7 | 43 | 49 |  | C | 8 |
| 269 | E |  | C | 8 | 45 | 47 |  | C | 4 |
| 270 | V |  | C | 6 | 46 | 49 |  | C | 3 |
| 271 | Y |  | C | 4 | 38 | 58 |  | C | 3 |
| 272 | D |  | C | 2 | 35 | 62 |  | C | 5 |
| 273 | T |  | C | 3 | 30 | 66 |  | C | 8 |
| 274 | P |  | C | 3 | 28 | 69 |  | C | 9 |
| 275 | P |  | C | 4 | 34 | 62 |  | C | 9 |
| 276 | M |  | C | 4 | 39 | 57 |  | C | 9 |
| 277 | A |  | C | 4 | 47 | 48 |  | C | 9 |
| 278 | V |  | C | 3 | 44 | 53 |  | C | 8 |
| 279 | K |  | C | 3 | 36 | 60 |  | C | 8 |
| 280 | G |  | C | 3 | 23 | 74 |  | C | 9 |
| 281 | P |  | C | 2 | 15 | 83 |  | C | 9 |
| 282 | N |  | C | 1 | 10 | 89 |  | C | 9 |
| 283 | G |  | C | 1 | 10 | 89 |  | C | 9 |
| 284 | R |  | C | 2 | 15 | 84 |  | C | 9 |
| 285 | D |  | C | 2 | 27 | 71 |  | C | 9 |
| 286 | P |  | C | 3 | 43 | 54 |  | C | 9 |
| 287 | L |  | E | 3 | 57 | 39 |  | C | 9 |
| 288 | L |  | E | 3 | 59 | 38 |  | C | 9 |
| 289 | D |  | E | 3 | 63 | 34 |  | C | 8 |
| 290 | V |  | E | 3 | 64 | 33 |  | C | 2 |
| 291 | Y |  | E | 3 | 49 | 48 |  | C | 1 |
| 292 | D |  | C | 2 | 43 | 56 |  | C | 4 |
| 293 | V |  | C | 3 | 31 | 66 |  | C | 7 |
| 294 | P |  | C | 3 | 18 | 79 |  | C | 9 |
| 295 | P |  | C | 6 | 19 | 75 |  | C | 9 |
| 296 | S |  | C | 8 | 20 | 72 |  | C | 9 |
| 297 | V |  | C | 14 | 31 | 55 |  | C | 8 |
| 298 | E |  | C | 18 | 40 | 42 |  | C | 5 |
| 299 | K |  | E | 23 | 43 | 34 |  | C | 5 |
| 300 | G |  | E | 16 | 52 | 32 |  | C | 8 |
| 301 | L |  | E | 11 | 56 | 33 |  | C | 8 |
| 302 | L |  | E | 9 | 53 | 37 |  | C | 8 |
| 303 | S |  | E | 9 | 53 | 38 |  | C | 9 |
| 304 | S |  | C | 9 | 45 | 46 |  | C | 9 |
| 305 | S |  | C | 5 | 42 | 52 |  | C | 9 |
| 306 | H |  | E | 5 | 53 | 42 |  | C | 9 |
| 307 | H |  | E | 3 | 56 | 41 |  | C | 9 |
| 308 | S |  | E | 2 | 55 | 42 |  | C | 8 |
| 309 | V |  | E | 2 | 57 | 42 |  | C | 6 |
| 310 | Y |  | E | 1 | 54 | 44 |  | C | 6 |
| 311 | D |  | E | 1 | 59 | 41 |  | C | 6 |
| 312 | V |  | C | 1 | 43 | 56 |  | C | 6 |
| 313 | P |  | C | 1 | 29 | 70 |  | C | 9 |
| 314 | P |  | C | 1 | 22 | 77 |  | C | 9 |
| 315 | S |  | C | 2 | 31 | 67 |  | C | 9 |
| 316 | V |  | C | 1 | 43 | 55 |  | C | 9 |
| 317 | S |  | C | 2 | 38 | 60 |  | C | 9 |
| 318 | K |  | C | 2 | 38 | 60 |  | C | 8 |
| 319 | D |  | C | 2 | 28 | 71 |  | C | 9 |
| 320 | V |  | C | 2 | 21 | 77 |  | C | 9 |
| 321 | P |  | C | 2 | 15 | 83 |  | C | 9 |
| 322 | D |  | C | 2 | 18 | 80 |  | C | 9 |
| 323 | G |  | C | 4 | 20 | 76 |  | C | 9 |
| 324 | P |  | C | 8 | 28 | 64 |  | C | 9 |
| 325 | L |  | C | 11 | 36 | 53 |  | C | 9 |
| 326 | L |  | C | 11 | 41 | 48 |  | C | 8 |
| 327 | R |  | C | 10 | 45 | 45 |  | C | 8 |
| 328 | E |  | C | 12 | 42 | 46 |  | C | 8 |
| 329 | E |  | C | 11 | 38 | 51 |  | C | 6 |
| 330 | T |  | C | 10 | 30 | 60 |  | C | 4 |
| 331 | Y |  | C | 7 | 24 | 69 |  | C | 5 |
| 332 | D |  | C | 3 | 19 | 77 |  | C | 8 |
| 333 | V |  | C | 6 | 17 | 77 |  | C | 9 |
| 334 | P |  | C | 3 | 18 | 79 |  | C | 9 |
| 335 | P |  | C | 3 | 17 | 81 |  | C | 9 |
| 336 | A |  | C | 3 | 17 | 80 |  | C | 9 |
| 337 | F |  | C | 3 | 18 | 79 |  | C | 9 |
| 338 | A |  | C | 2 | 17 | 81 |  | C | 9 |
| 339 | K |  | C | 2 | 12 | 86 |  | C | 9 |
| 340 | P |  | C | 1 | 10 | 89 |  | C | 9 |
| 341 | K |  | C | 1 | 9 | 91 |  | C | 9 |
| 342 | P |  | C | 0 | 6 | 94 |  | C | 9 |
| 343 | F |  | C | 1 | 6 | 93 |  | C | 9 |
| 344 | D |  | C | 1 | 6 | 92 |  | C | 9 |
| 345 | P |  | C | 2 | 9 | 89 |  | C | 9 |
| 346 | T |  | C | 4 | 11 | 85 |  | C | 9 |
| 347 | R |  | C | 4 | 18 | 78 |  | C | 9 |
| 348 | H |  | C | 8 | 27 | 65 |  | C | 9 |
| 349 | P |  | C | 8 | 46 | 47 |  | C | 9 |
| 350 | L |  | E | 8 | 50 | 42 |  | C | 7 |
| 351 | I |  | E | 6 | 54 | 40 |  | C | 5 |
| 352 | L |  | E | 3 | 61 | 36 |  | C | 6 |
| 353 | A |  | C | 2 | 41 | 57 |  | C | 8 |
| 354 | A |  | C | 1 | 14 | 85 |  | C | 9 |
| 355 | P |  | C | 0 | 6 | 94 |  | C | 9 |
| 356 | P |  | C | 0 | 3 | 97 |  | C | 9 |
| 357 | P |  | C | 0 | 2 | 98 |  | C | 9 |
| 358 | D |  | C | 0 | 2 | 98 |  | C | 9 |
| 359 | S |  | C | 2 | 4 | 94 |  | C | 9 |
| 360 | P |  | C | 5 | 6 | 88 |  | C | 9 |
| 361 | A |  | C | 8 | 13 | 80 |  | C | 9 |
| 362 | A |  | C | 9 | 22 | 69 |  | C | 8 |
| 363 | E |  | C | 7 | 33 | 60 |  | C | 9 |
| 364 | D |  | C | 6 | 46 | 49 |  | C | 8 |
| 365 | V |  | C | 0 | 49 | 51 |  | C | 7 |
| 366 | Y |  | C | 2 | 46 | 51 |  | C | 7 |
| 367 | D |  | C | 1 | 32 | 67 |  | C | 6 |
| 368 | V |  | C | 1 | 23 | 76 |  | C | 8 |
| 369 | P |  | C | 0 | 12 | 88 |  | C | 9 |
| 370 | P |  | C | 0 | 7 | 93 |  | C | 9 |
| 371 | P |  | C | 0 | 6 | 94 |  | C | 9 |
| 372 | A |  | C | 0 | 8 | 92 |  | C | 9 |
| 373 | P |  | C | 0 | 13 | 86 |  | C | 9 |
| 374 | D |  | C | 1 | 17 | 82 |  | C | 9 |
| 375 | L |  | C | 1 | 24 | 74 |  | C | 9 |
| 376 | Y |  | C | 1 | 22 | 76 |  | C | 8 |
| 377 | D |  | C | 1 | 23 | 76 |  | C | 9 |
| 378 | V |  | C | 2 | 28 | 70 |  | C | 9 |
| 379 | P |  | C | 1 | 22 | 77 |  | C | 9 |
| 380 | P |  | C | 1 | 24 | 75 |  | C | 9 |
| 381 | G |  | C | 1 | 27 | 72 |  | C | 9 |
| 382 | L |  | C | 1 | 27 | 72 |  | C | 9 |
| 383 | R |  | C | 1 | 27 | 72 |  | C | 9 |
| 384 | R |  | C | 1 | 26 | 73 |  | C | 9 |
| 385 | P |  | C | 0 | 21 | 79 |  | C | 9 |
| 386 | G |  | C | 0 | 19 | 81 |  | C | 9 |
| 387 | P |  | C | 1 | 20 | 79 |  | C | 9 |
| 388 | G |  | C | 1 | 29 | 71 |  | C | 9 |
| 389 | T |  | C | 2 | 38 | 61 |  | C | 9 |
| 390 | L |  | C | 3 | 44 | 53 |  | C | 7 |
| 391 | Y |  | C | 5 | 44 | 52 |  | C | 7 |
| 392 | D |  | C | 4 | 48 | 48 |  | C | 7 |
| 393 | V |  | C | 5 | 45 | 50 |  | C | 9 |
| 394 | P |  | C | 7 | 39 | 54 |  | C | 9 |
| 395 | R |  | C | 7 | 37 | 56 |  | C | 8 |
| 396 | E |  | C | 9 | 34 | 57 |  | C | 8 |
| 397 | R |  | C | 7 | 34 | 59 |  | C | 8 |
| 398 | V |  | C | 4 | 36 | 60 |  | C | 8 |
| 399 | L |  | C | 4 | 39 | 57 |  | C | 9 |
| 400 | P |  | C | 3 | 33 | 64 |  | C | 9 |
| 401 | P |  | C | 5 | 31 | 63 |  | C | 9 |
| 402 | E |  | C | 7 | 28 | 65 |  | C | 7 |
| 403 | V |  | C | 7 | 34 | 60 |  | C | 6 |
| 404 | A |  | C | 5 | 42 | 53 |  | C | 8 |
| 405 | D |  | C | 4 | 42 | 54 |  | C | 9 |
| 406 | G |  | E | 4 | 52 | 44 |  | C | 9 |
| 407 | S |  | E | 5 | 60 | 35 |  | C | 9 |
| 408 | V |  | E | 6 | 62 | 32 |  | C | 8 |
| 409 | V |  | E | 6 | 65 | 29 |  | C | 8 |
| 410 | D |  | E | 5 | 70 | 25 |  | C | 9 |
| 411 | D |  | E | 3 | 71 | 26 |  | C | 9 |
| 412 | G |  | E | 2 | 69 | 28 |  | C | 8 |
| 413 | V |  | C | 1 | 41 | 58 |  | C | 0 |
| 414 | Y |  | C | 0 | 12 | 88 |  | C | 4 |
| 415 | A |  | C | 0 | 2 | 97 |  | C | 4 |
| 416 | V |  | C | 0 | 0 | 100 |  | C | 5 |
| 417 | P |  | C | 0 | 0 | 100 |  | C | 9 |
| 418 | P |  | C | 0 | 0 | 100 |  | C | 9 |
| 419 | P |  | C | 0 | 0 | 100 |  | C | 9 |
| 420 | A |  | C | 0 | 0 | 100 |  | C | 9 |
